# Gene editing in clinical isolates of *Candida parapsilosis* using CRISPR/Cas9

**DOI:** 10.1038/s41598-017-08500-1

**Published:** 2017-08-14

**Authors:** Lisa Lombardi, Siobhán A. Turner, Fang Zhao, Geraldine Butler

**Affiliations:** School of Biomolecular and Biomedical Science, Conway Institute, University College Dublin, Belfield, Dublin 4, Ireland

## Abstract

*Candida parapsilosis* is one of the most common causes of candidiasis, particularly in the very young and the very old. Studies of gene function are limited by the lack of a sexual cycle, the diploid genome, and a paucity of molecular tools. We describe here the development of a plasmid-based CRISPR-Cas9 system for gene editing in *C*. *parapsilosis*. A major advantage of the system is that it can be used in any genetic background, which we showed by editing genes in 20 different isolates. Gene editing is carried out in a single transformation step. The *CAS9* gene is expressed only when the plasmid is present, and it can be removed easily from transformed strains. There is theoretically no limit to the number of genes that can be edited in any strain. Gene editing is increased by homology-directed repair in the presence of a repair template. Editing by non-homologous end joining (NHEJ) also occurs in some genetic backgrounds. Finally, we used the system to introduce unique tags at edited sites.

## Introduction

*Candida parapsilosis* is a pathogenic yeast, and is one of the five most common species associated with candidiasis^1^. Infection is particularly common in infants less than 1 year old^2^. *C*. *parapsilosis* is found on the hands of healthcare workers, and transmission has been associated with outbreaks of infection across the globe^3–6^.

*C*. *parapsilosis* is a member of the CUG-Ser clade, species that encode CUG as serine rather than leucine^7, 8^. This clade includes diverse species assigned to the Debaryomycetaceae, the Metschnikowiaceae and the Cephaloascaceae families^9, 10^. Many have important biotechnology applications, including the ability to utilize xylose^7, 11, 12^. The Candida/Lodderomyces sub-clade includes at least 30 described species, some of which are human fungal pathogens^13^. Most Candida/Lodderomyces species have no sexual cycle, or at best a parasexual cycle; spore formation has been described only in *Lodderomyces elongisporus* ^14^, and meiosis has never been observed in the *Candida* species of this clade. Diploid cells of *C*. *albicans*, *Candida dubliniensis* and *Candida tropicalis* have been shown to mate and form tetraploids^15–17^. In *C*. *albicans*, cells revert to diploidy via concerted chromosome loss^18^. All characterized isolates of *C*. *parapsilosis* contain the same Mating Type-like Locus (*MTL* **a**), and mating has never been observed^19–21^.

The diploid nature, the lack of a sexual cycle, and the use of the CUG codon mean that generating gene disruptions in *Candida* species is an arduous process. Manipulation of *Candida* species is further hampered by the lack of dominant selectable markers^22^. Roemer *et al*.^23^ and Reuss *et al*.^24^ constructed codon-modified versions of the *Escherichia coli SAT1* gene, allowing the use of nourseothricin resistance as a selectable marker in *C*. *albicans*. Reuss *et al*.^24^ combined the *Candida*-optimized *SAT1* gene, flanked by recombination sites, into a cassette together with a regulatable site-specific recombinase. Sequences from upstream and downstream of the target gene are introduced at the ends of the cassette by cloning or by PCR. The construct is then used to replace one allele of the target gene in *C*. *albicans* by homologous recombination. Inducing expression of the recombinase facilitates recycling of the nourseothricin resistance marker, so that the cassette (or a similar cassette) can be used to delete the second allele. The “*SAT1* flipper” system has been further adapted for use in other CUG clade species, including *C*. *parapsilosis* ^25–27^ and *Meyerozyma guilliermondii* ^28^. The *SAT1* flipper cassette is a very powerful technique, and it has been used to delete up to eight genes in the same strain of *C*. *albicans* ^29^. However, deleting multiple genes can be a long process, as the *SAT1* cassette must be recycled following deletion of each allele.

Noble *et al*.^30^ developed a faster gene deletion method by constructing strains of *C*. *albicans* that were auxotrophic for up to three markers. Target genes are deleted by sequentially replacing each allele with a different marker, ultimately restoring the strain to prototrophy. Marker constructs are generated by fusion PCR, streamlining the generation of gene deletions. This method has been used for high-throughput gene deletion analysis both in *C*. *albicans* ^31^ and *C*. *parapsilosis* ^32^. However, generating a homozygous gene deletion remains a two-step process, as each allele is usually independently targeted. The method also requires an auxotrophic parent, limiting its application to a small number of laboratory strains. Finally, it is not possible to target multiple genes using this approach, as the auxotrophic marker genes cannot be re-used.

Many of the drawbacks outlined for the existing deletion approaches were recently addressed by adapting the clustered regularly interspaced short palindromic repeats (CRISPR)-Cas9 system method for use in *C*. *albicans* ^33–37^. CRISPR/Cas was originally identified as part of the adaptive immune response of archaea and bacteria against viral DNA^38^. The Cas endonuclease is targeted to the phage DNA by small RNAs, where it introduces double stranded breaks. Most adaptations of the system for gene editing in eukaryotes use Cas9 from *Streptococcus pyogenes* and a single synthetic guide RNA (sgRNA)^39, 40^. The only additional requirement is that the target site on the genome is followed by an “NGG” protospacer adjacent motif (PAM). The sgRNA comprises both a 20 bp RNA sequence complementary to a target DNA adjacent to the NGG, and a longer trans-activating RNA, which facilitates binding to Cas9. The endonuclease induces a double stranded break (DSB) at the target site, which is repaired either by non-homologous end-joining (NHEJ), or homology-directed repair (HDR). NHEJ is error-prone, and usually introduces small deletions or insertions^40^. HDR combined with linear homologous molecules of DNA (repair template) can be used to introduce specific sequences at the break site^40^.

In the first application of CRISPR/Cas9 in *C*. *albicans*, Vyas *et al*.^33^ designed a codon-modified version of Cas9 that is integrated into the *C*. *albicans* genome. The sgRNA directing Cas9 to the target gene is expressed from an RNA polymerase III promoter, and is also integrated in the genome, either together with Cas9, or at a different site. The system was used together with repair templates to introduce premature stop codons in two alleles of the target gene, thus generating a homozygous mutant strain in a single transformation. Concerns that the prolonged presence of *CAS9* in the *C*. *albicans* genome could lead to long-term detrimental side effects, such as off-target effects, were addressed by Min *et al*.^34^. They showed that *CAS9* and the sgRNA could be transiently expressed, thus eliminating the need for integration^34^. The *CAS9* and sgRNA constructs are co-transformed with a repair template that either confers resistance to nourseothricin, or encodes amino acid biosynthesis proteins in combination with suitable auxotrophic parental strains. The selectable markers replace the open reading frames of the target genes. Huang *et al*.^35^ further modified the system by surrounding the selectable markers with direct repeats, enabling CRISPR-directed marker recycling. Two marker genes can be used to sequentially delete three or more genes in the same strain^35^. All constructs are generated by PCR, without the need for cloning.

The CRISPR methods developed by Vyas *et al*.^33^, Min *et al*.^34^ and Huang *et al*.^35^ greatly improved the state-of-the-art in *C*. *albicans* genetics. However, most applications require auxotrophic strains, which limits their use to specially engineered isolates of *C*. *albicans*. This was addressed by Nguyen *et al*.^37^ who adapted the *SAT1* flipper system to enable CRISPR-based sequential gene deletion in any nourseothricin-sensitive strain of *C*. *albicans*. In the HIS-FLP system, an FRT flanked cassette carrying the *SAT1* selectable marker, *FLP* recombinase, *CAS9* and the sgRNA is integrated at one allele of *HIS1*. Cas9-mediated cleavage is repaired by HDR using a short repair template that is homologous to sequences upstream and downstream regions of the target gene, resulting in a homozygous deletion. Induction of the *FLP* recombinase triggers the excision of the cassette, leaving an FRT site within *HIS1*, which inactivates one of the alleles. The alternative LEUpOUT systems can be used for iterative editing of several genes. The *CAS9*/*SAT1*/sgRNA cassettes are surrounded by repeated sequences derived from *LEU2*, and are integrated at one *LEU2* allele. Following the gene editing event, the cassette can be recycled by selecting for recombination between the repeats, which restores a functional *LEU2* allele. All steps are PCR-based and cloning free, and no selectable markers are left in the genome. However, to use LEUpOUT the parental strain must contain only one functional *LEU2* allele. Nguyen *et al*.^37^ also used CRISPR-mediated cleavage to complement the deleted genes.

Ng *et al*.^36^ showed that the efficiency of CRISPR-mediated gene editing in *C*. *albicans* can be improved by increasing the expression of the guide RNA^36^. They achieved this by replacing the RNA polymerase III promoter with an RNA polymerase II promoter. Excision of the sgRNA from the polymerase II transcript is facilitated by flanking it with ribozyme or tRNA sequences. Both *CAS9* and sgRNA constructs are integrated in the genome of an auxotrophic *C*. *albicans* strain.

There are therefore now several CRISPR-based methods that can be used for editing or deleting genes in C. *albicans*. However, they cannot be used in other *Candida* species without substantial alterations^41^. Norton *et al*.^41^ adapted the transient expression system developed by Min *et al*.^34^ for use in *Clavispora lusitaniae* by replacing the promoters driving expression of *CAS9* and the sgRNA. Grahl *et al*.^42^ used a different approach, purifying Cas9 protein and CRISPR RNAs and introducing the product directly into the cell by electroporation, instead of expressing the genes. Grahl *et al*.^42^ showed that this system can be used to edit genes in multiple species, including *C*. *lusitaniae*, *C*. *glabrata* and *Candida auris*. However, both these approaches incorporate the *SAT1* gene in the repair templates, which is used for selection, and which remains at the target site following the gene deletion event.

Here we describe a plasmid-based CRISPR method that can be applied in *C*. *parapsilosis*. Because *CAS9* and the guide RNA are maintained on a plasmid that replicates in *C*. *parapsilosis* ^43^, the system can be used in any strain, including clinical isolates. Only one dominant selectable marker is used, and the plasmid is lost following the editing event. No selectable markers remain in the genome, which enables sequential editing of any number of target genes using the same marker without recycling. Guide RNAs are cloned between two ribozymes with expression driven from an RNA polymerase II promoter^44^. The system is highly efficient, yielding up to 100% efficiency across a panel of 20 clinical isolates.

## Results

### Developing a CRISPR system for *C*. *parapsilosis*

**Figure 1:**
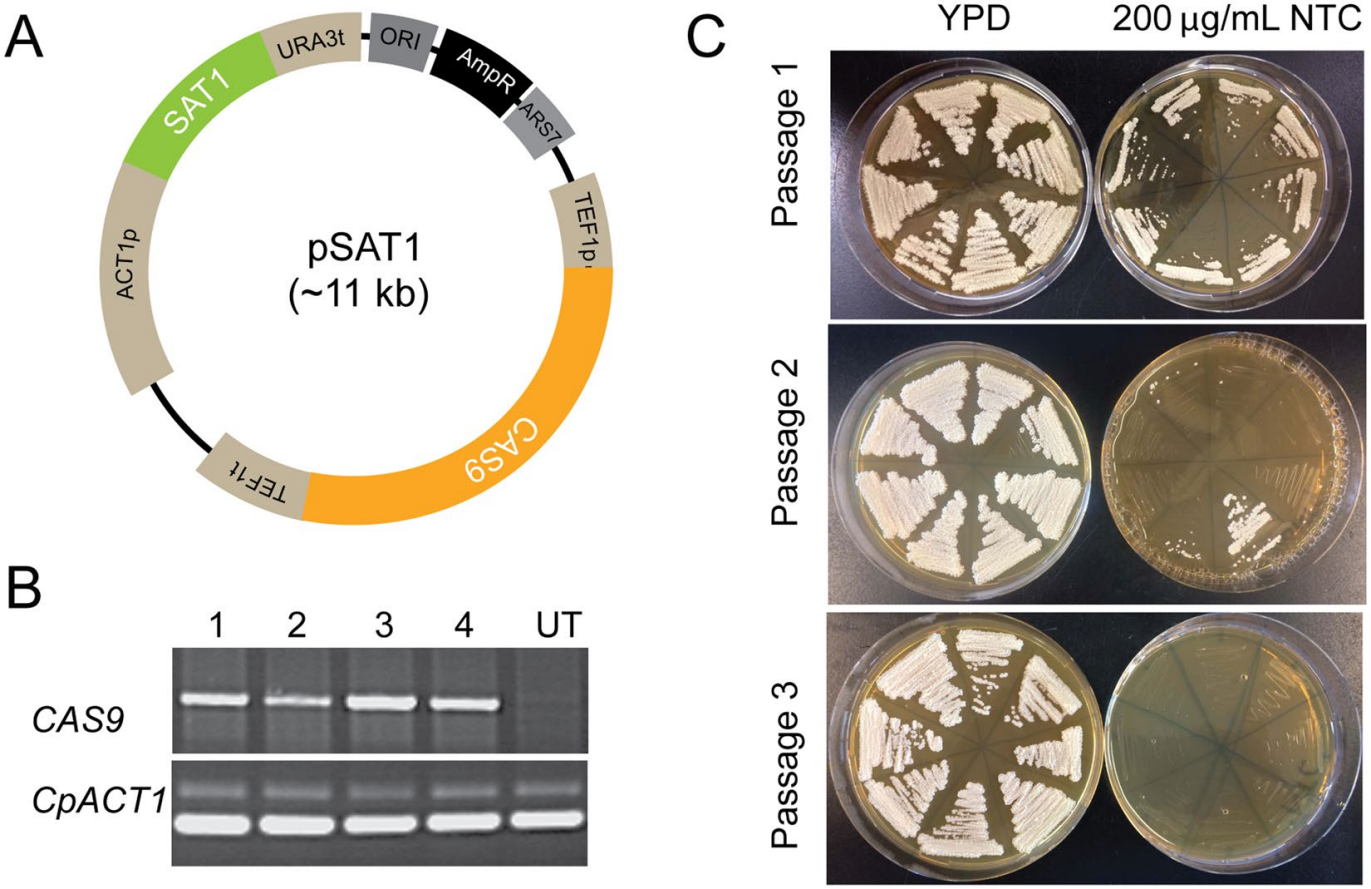
Autonomously replicating plasmids in *C*. *parapsilosis*. (**A**) pSAT1 was constructed by cloning a codon-optimized version of *CAS9* between the promoter and terminator sequences of *TEF1* from *C*. *parapsilosis* in a pUC57-based plasmid. *SAT1* (nourseothricin resistance) expressed from the *C*. *albicans ACT1* promoter was isolated from pSFS2A^58^, and *ARS7*, an autonomously replicating sequence from *C*. *parapsilosis* ^43^, was isolated from pGIZI. (**B**) *CAS9* is expressed in *C*. *parapsilosis* cells transformed with pSAT1. RNA was isolated from four transformants and from one untransformed culture (UT). Expression of *CAS9* and *ACT1* was measured by RT-PCR. (**C**) pSAT1 is easily lost. Transformed cells were patched to YPD plates without nourseothricin (NTC) for 48 h, and then streaked on YPD and YPD + NTC. Colonies from YPD were repatched after 48 hr. All transformants lost nourseothricin resistance after just two passages.

We first synthesized a codon-optimized version of *CAS9*, eliminating all CTG codons and incorporating a Nuclear Localization Sequence (NLS) (Supplementary Fig. S1). This sequence was cloned into a standard cloning plasmid flanked by the *TEF1* regulatory sequences amplified from *C*. *parapsilosis* genomic DNA. Autonomously replicating plasmids are not commonly used in *Candida* species^22^. However, Nosek *et al*.^43^ described the identification of some sequences that promote autonomous replication in *C*. *parapsilosis*. We adapted one of these autonomous replication sequences (ARS7). This sequence was originally obtained by screening fragments from a *Sau*3AI digestion of *C*. *parapsilosis* genomic DNA^43^. Now that the genome sequence of *C*. *parapsilosis* is available^19^ we know that the “ARS” results from ligating two fragments that are derived from different places on chromosomes 4 and 5. ARS7 and the *SAT1* nourseothricin resistance gene under the control of the *CaACT1* promoter and the *CaURA3* terminator were cloned into the plasmid encoding Cas9 to generate pSAT1 (Fig. 1A). This plasmid confers resistance to nourseothricin when transformed into *C*. *parapsilosis*, and resistance is lost after just two passages in the absence of selection (Fig. 1). The plasmid therefore does not integrate into the genome. Transcription of *CAS9* was demonstrated by RT-PCR (Fig. 1B).

**Figure 2:**
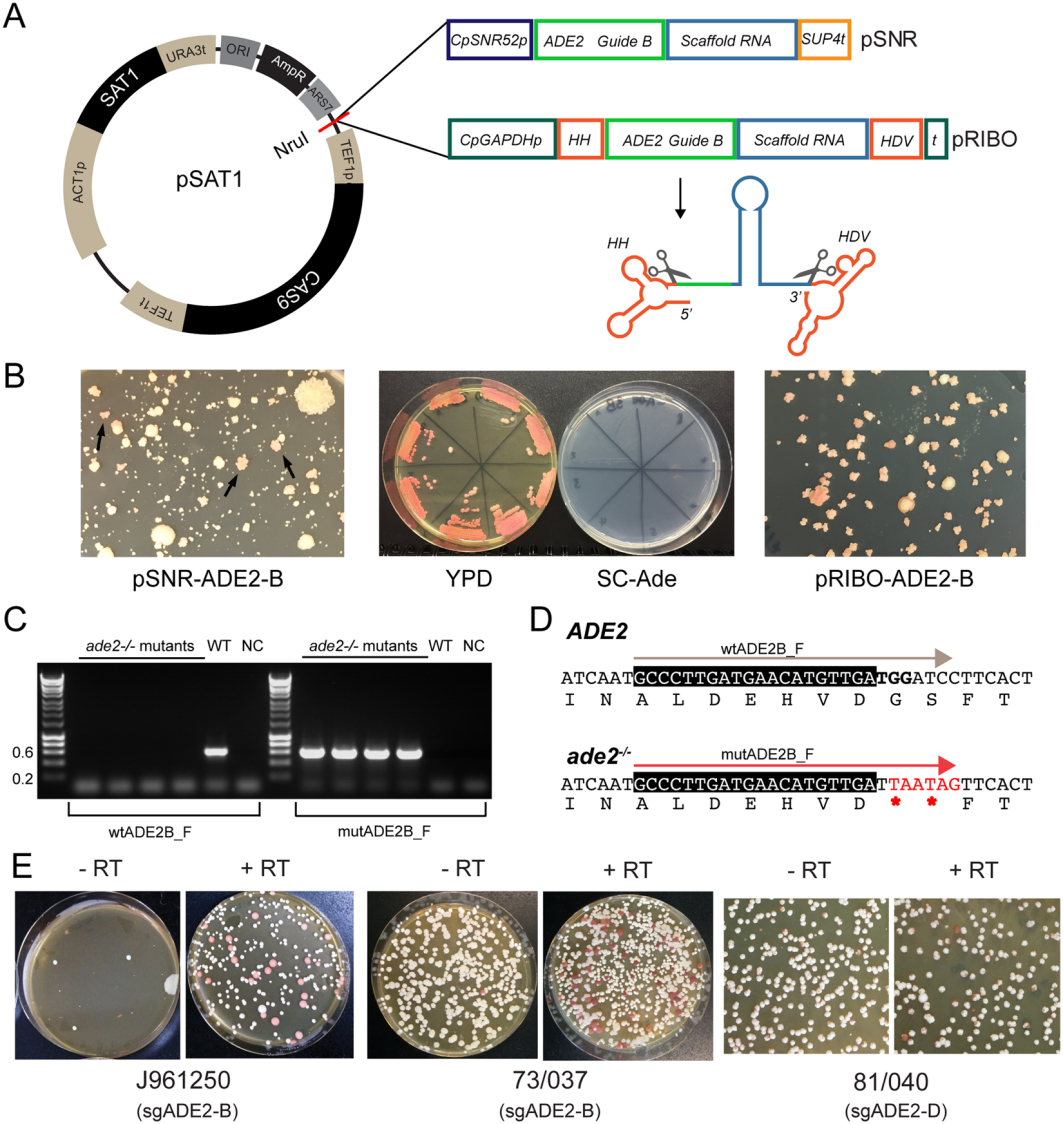
Editing *ADE2* in *C*. *parapsilosis* using CRISPR. (**A**) Two constructs were generated by inserting one of two cassettes expressing a guide RNA targeting *ADE2* at the NruI site of pSAT1. In the first cassette (pSNR), expression of the ADE2-B sgRNA is driven from the *SNR52* promoter (dark blue), generating pSNR-ADE2-B. In the second cassette (pRIBO) the ADE2-B sgRNA is surrounded by two ribozymes (HH and HDV in red), and expression is driven from the *GAPDH* promoter (teal), generating pRIBO-ADE2-B. The scaffold sequence is shown in blue, the targeting sequence in green, the *SUP4* terminator sequence in orange, and the *GAPDH* terminator in teal. Folding and cleavage of the guide RNA at the ribozyme sequences is shown below the pRIBO cassette. (**B**) Transformation of *C*. *parapsilosis* CLIB214 with pSNR-ADE2-B and a repair template (Supplementary methods) produced nourseothricin-resistant colonies of different sizes, including some that turned light pink after 3–5 days (black arrows, left). The pink color is more noticeable when streaked on YPD plates (middle). All pink colonies failed to grow in the absence of adenine (SC-ade). Transformation with pRIBO-ADE2-B produced colonies of more uniform size (right). (**C**) Pink colonies transformed with pSNR-ADE2-B were screened by PCR using one common primer (ADE2_REV) and one primer specific for either the wildtype (wtADE2B_F) or the mutant (mutADE2B_F) sequence, shown in gray and red in (**D**). A 631 bp PCR product was amplified from the mutant colonies only when the mutADE2B_F primer was used, and from the wildtype (WT) only when the wtADE2B_F was used. NC = no DNA. (**D**) Sequence of the *ADE2* locus before and after the CRISPR-Cas9 mediated mutation. Following Cas9 induced cleavage, homologous recombination with a repair template results in the insertion of two stop codons in frame (shown in red) replacing the PAM site, thus disrupting the gene function. The gRNA sequence is highlighted with a black box, and the PAM sequence is shown in bold. The primers used in (**C**) are indicated above the sequences. (**E**) Transformation of clinical isolates of *C*. *parapsilosis* with the ribozyme plasmids pRIBO-ADE2-B (strains J961250 and 73/037) or with pRIBO-ADE2-D (strain 81/040). RT = repair template.

In most CRISPR-based methods, including the commonly used systems in *C*. *albicans*, expression of the short guide RNAs is driven from the RNA polymerase III promoter *SNR52* ^33–35, 37^. We tried to develop a similar system, using the putative promoter from the *C*. *parapsilosis* homolog of *SNR52* ^45^. We first targeted the *ADE2* gene, because disruptants are easily identified by the formation of pink colonies on YPD media. A 20 bp-long synthetic guide RNA (guide B, +130 bp downstream from the ATG) was designed by using the EuPaGDT web tool^46^. The SNR-ADE2-B cassette, including a terminator sequence from *S*. *cerevisiae SUP4* ^47^, was inserted into pSAT1 in a two-step process (see Methods), generating pSNR-ADE2-B (Fig. 2A). This was transformed into *C*. *parapsilosis* CLIB214, with or without a repair template designed both to introduce two stop codons and to mutate the target PAM site (Fig. 2B–D). In the presence of the repair template, pink adenine auxotrophs were observed, with a frequency ranging from 10 to 50% (Fig. 2B). However, the nourseothricin (NTC) resistant transformants are highly variable in size. We used colony PCR to discriminate between wild-type cells and cells that had incorporated the repair template (Fig. 2C). Sequencing of two representative colonies confirmed that the repair template had been incorporated (Fig. 2D). CRISPR-based gene editing can therefore be used to disrupt both alleles at a single locus in *C*. *parapsilosis*.

To improve the efficiency of gene editing, we adapted a method used in other fungi^36, 44, 48, 49^ and recently applied in *C*. *albicans* ^36^, whereby sgRNAs are expressed from a pol II promoter (*GAPDH* promoter from *C*. *parapsilosis*), flanked by a hammerhead (HH) ribozyme and a hepatitis delta virus (HDV) ribozyme. Following transcription, self-cleavage by the two ribozymes releases the functional sgRNA (Fig. 2A). Transformation of *C*. *parapsilosis* CLIB214 with pRIBO-ADE2-B plasmid yielded more uniform colonies than the transformants obtained using the pSNR-ADE2-B plasmid (Fig. 2B), and 80–100% were pink. To test the reproducibility of the CRISPR system, we replaced the gRNA with a second sequence (sgADE2-D, Supplementary Table S1) also targeting *ADE2* (+451 bp from ATG). Once again, transformation of *C*. *parapsilosis* CLIB214 with pRIBO-ADE2-D and a new repair template (Supplementary Table S1) generated pink colonies (Table 1) with high efficiency (94–100%). Two representative colonies from each transformation were sequenced, confirming the presence of the expected mutation at the *ADE2* locus. Similar to pSAT1, pSNR and pRIBO plasmids are easily lost when cells are grown on non-selective media (Supplementary Fig. S2). On rare occasions, the plasmid may integrate into the genome and we identified one possible example using pRIBO-ADE2-D (Supplementary Fig. S2).

**Table 1:** Efficiency of CRISPR-based editing of *ADE2* in multiple isolates of *C*. *parapsilosis*.

Strain	sgRNA	% pink transformants^a^			
		−RT^c^		+RT^c^	
		Exp 1	Exp 2	Exp 1	Exp 2
CLIB214^d^	sgADE2-B	0%	0%	80%	100%
CLIB214^d^	sgADE2-D	0%	0%	94%	100%
103^d^	sgADE2-B	0%	12%	50%	80%
02-203^d^	sgADE2-B	0%	0%	87%	94%
73/037	sgADE2-B	11%	0%	5%	17%
73/107^d^	sgADE2-B	4%	0%^b^	4%	16%
74/046	sgADE2-B	0%	0%^b^	30%	4%
81/040	sgADE2-B	0%	0%	0%	0%
81/042^d^	sgADE2-B	0%	0%^b^	8%	14%
81/253^d^	sgADE2-B	0%	1%	12%	35%
90–137	sgADE2-B	20%	10%	37%	67%
CDC165^d^	sgADE2-B	20%	1%	0%	87%
CDC167	sgADE2-B	0%	1%	15%	14%
CDC173	sgADE2-B	0%	3%	13%	10%
CDC177^d^	sgADE2-B	4%	0%^b^	3%	24%
CDC179	sgADE2-B	0%	11%	35%	25%
CDC317	sgADE2-B	0%	13%	16%	16%
J931058	sgADE2-B	0%	31%	100%	77%
J931845	sgADE2-B	0%	0%^b^	28%	13%
J950218	sgADE2-B	11%	1%	14%	7%
J951066	sgADE2-B	0%	14%	11%	85%
J960578^d^	sgADE2-B	0%	4%	25%	13%
J961250	sgADE2-B	0%	0%^b^	29%	19%

^a^Transformation efficiencies from two experiments performed by two people. 81/040 and CLIB214 were transformed several times and the results from two experiments are shown.

^b^<1% pink colonies.

^c^RT = repair template.

^d^Isolates sequenced to confirm mutation (Fig. S3).

### Editing clinical isolates of *C*. *parapsilosis*

One major advantage of a plasmid-based CRISPR system with a dominant selectable marker is that it can be used to edit genes in any nourseothricin-sensitive isolate, and not only in engineered laboratory strains. We tested this by transforming 20 *C*. *parapsilosis* strains (Table 1) with the ribozyme construct expressing sgADE2-B, in the presence and absence of a repair template (Fig. 2E). 19 of the transformed strains yielded pink *ADE2* disruptants when the repair template was provided (Table 1). The efficiency of the transformation varied considerably; the number of transformants obtained ranged from less than 10 to more than 1,000, depending on the experimenter and the genetic background (some examples are shown in Fig. 2E). The efficiency of gene editing also varied (Table 1, Fig. 2E). Unlike *C*. *parapsilosis* CLIB214, transformation of many of the clinical isolates yielded pink colonies even in the absence of a repair template (Table 1). We sequenced the edited genes from eight isolates generated in the presence of repair template, and four without the repair template (Table 1, Supplementary Fig. S3). When the repair template was present the expected mutations were observed, indicating that homology dependent repair has occurred (Fig. 2D, Supplementary Fig. S3). In the absence of the repair template, we observed insertions and deletions adjacent to the Cas9 cleavage site, resulting in frameshifts and often in premature stop codons downstream (Supplementary Fig. S3). For the final strain, *C*. *parapsilosis* 81/040, which produced no pink colonies using sgADE2-B, we attempted to generate adenine auxotrophs by transforming with sgADE2-D with and without the repair template. Pink colonies were obtained in both cases, although mixed populations of white and pink cells were common (Fig. 2E). Somewhat surprisingly, sequencing of six adenine auxotrophs from three independent experiments transformed using the repair template showed that the template was not incorporated in this strain, and instead there was an insertion of one additional base at the Cas9 cleavage site (Supplementary Fig. S3).

### Editing additional genes

**Figure 3:**
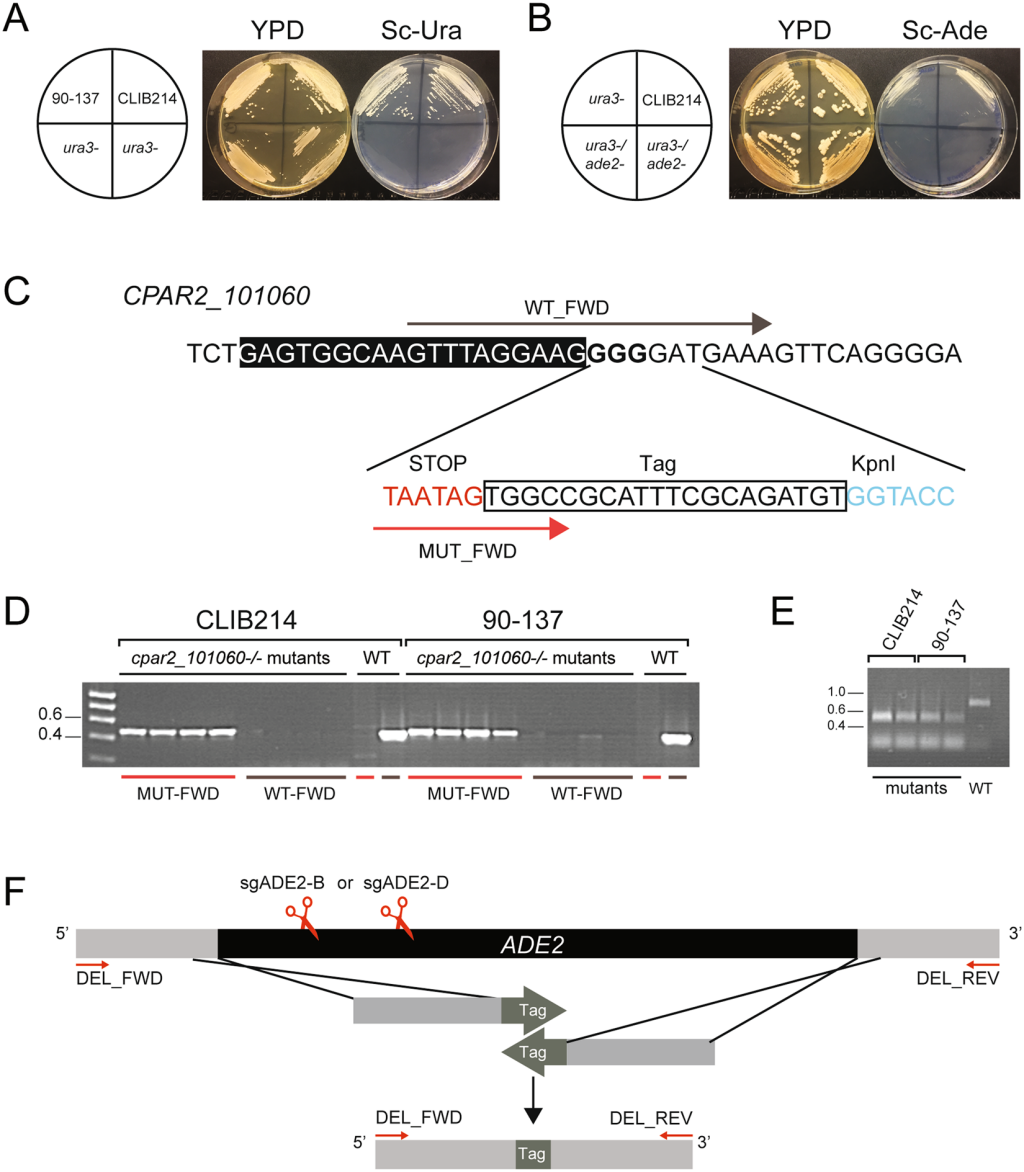
Editing multiple genes in *C*. *parapsilosis*. (**A**) The *URA3* gene in two strains of *C*. *parapsilosis* (CLIB214 and 90–137) was edited using pRIBO-URA3-385 and the relevant repair template (Supplementary Table S1). The figure shows a representative uracil auxotroph from each genetic background. Incorporation of the repair template was confirmed by sequencing. (**B**) The *ADE2* gene was edited in one uracil auxotroph of *C*. *parapsilosis* CLIB214 (shown in panel A), using pRIBO-ADE2-D, generating strains that are pink on YPD, and fail to grow in the absence of uracil or adenine. Incorporation of the relevant repair template was confirmed by sequencing (Supplementary Fig. S3). (**C**) To edit *CPAR2_101060*, a repair template was designed including two stop codons, a unique 20 base pair tag, and a KpnI restriction site. The entire template is 108 base pairs. (**D**) Following transformation of two *C*. *parapsilosis* strains (CLIB214 and 90–137) colonies were screened by PCR using one common primer (CP101060_WT-R) and one primer specific for either the wild type (CP101060_WT_F, indicated here as WT-FWD) or the mutant (CP101060_MUT_F, indicated here as MUT-FWD) sequence, shown in brown and red in (**C**). A 458 bp PCR product was amplified from the mutant colonies only when the MUT_FWD primer was used, and from the wildtype (WT) only when the WT_FWD was used. (**E**) An 898 bp fragment was amplified using primers CP101060-F and CP101060_WT_R from two putative *CPAR2_101060* disruptants from each *C*. *parapsilosis* background, and from wildtype *C*. *parapsilosis* CLIB214. Only strains in which *CPAR2_101060* was edited were digested with KpnI. (**F**) To completely delete *ADE2*, a double stranded break was introduced using either sgADE2-B or sgADE2-D and a repair template including 40 bp from the 5′ and 3′ flanking regions and a unique 20 bp tag. Deletion mutants were verified by colony PCR using primers (DEL_FWD + REV) flanking the deleted region and this was confirmed by sequencing (Fig. S3).

We further investigated the adaptability of the CRISPR-based editing system by targeting additional genes. We first designed two sgRNAs directed against *URA3* (sgURA3_358 and sgURA3_195, Supplementary Table S1), and expressed them in the ribozyme-based plasmid. Transformation of *C*. *parapsilosis* strains CLIB214 and 90–137 with pRIBO-URA3-358 and the relevant repair template produced uracil auxotrophs at an efficiency of 60 and 37%, respectively (Fig. 3A, Supplementary Fig. S4). Incorporation of the repair template was confirmed by sequencing two colonies for each strain. Constructs incorporating guide RNA 195 did not generate any uracil auxotrophs in either of the strains.

Until this point, the target gene was chosen based on the ability to easily identify transformants in which both alleles had been disrupted, producing pink and/or auxotrophic colonies. To be truly useful, a gene editing system must function at a high efficiency even when there is no known phenotype. We therefore targeted *CPAR2_101060*, a transcription factor that was previously deleted by Holland *et al*.^32^ using the fusion PCR approach. A homozygous deletion of *CPAR2_101060* has no obvious phenotype. We also explored the possibility of including a unique barcode (tag), and a restriction site in the repair template (Fig. 3C). Transformation of two *C*. *parapsilosis* strains (CLIB214 and 90–137) in the presence of repair template yielded 4–30 colonies. No transformants were obtained without a repair template. Allele-specific PCR of four colonies from each strain showed that both alleles of *CPAR2_101060* had been edited in all transformants tested (Fig. 3D). We then amplified a region surrounding the repair template, and showed that DNA from all transformants, but not from the wild type strain, could be digested with KpnI (Fig. 3E). Finally, sequencing of this region amplified from one transformant of each strain confirmed that the repair template was incorporated as expected. We therefore demonstrate editing of a gene with no observable phenotype at very high efficiencies (in this case up to 100%), screening either by allele-specific PCR or by enzymatic digestion. We also show that the inclusion of a unique barcode in each edited strain is straightforward and does not reduce the efficiency of editing.

### Editing multiple genes in *C*. *parapsilosis*

In theory, our system should allow sequential editing of any number of genes using plasmids encoding different sgRNAs. We tested this by attempting to edit the *ADE2* gene in the *C*. *parapsilosis* CLIB214 mutant strain in which the *URA3* gene had already been disrupted. Two independent transformations with pRIBO-ADE2-D and the corresponding repair template were performed, generating 80–160 nourseothricin resistant transformants. All transformants failed to grow in absence of adenine, demonstrating that the efficiency of the homozygous *ade2* editing in the uracil auxotroph was 100% (Fig. 3B, Supplementary Fig. S4). The sequencing of both *ADE2* and *URA3* loci from one isolate confirmed that the repair templates had been incorporated as expected. There were no transformants in the absence of the repair template.

### Generating gene deletions

The repair templates shown in Figs 2–3C were designed to introduce stop codons and/or barcodes at the target site (gene editing). In other *Candida* species CRISPR/Cas9 has been used to delete or replace genes, by designing repair templates that incorporate sequences flanking the open reading frame^34, 36, 37, 41, 42^. We therefore tested the capacity of our system to delete, rather than edit, the *ADE2* gene in *C*. *parapsilosis*. A 100 bp repair template (RT_DEL) was constructed by primer extension, incorporating 40 bp sequences flanking the *ADE2* ORF and a unique 20 bp barcode sequence (Fig. 3F). Co-transforming *C*. *parapsilosis* 90–137 cells with pRIBO-ADE2-B or pRIBO-ADE2-D and RT_DEL generated pink *ADE2* deletion mutants at a frequency of 60–100%. One deletion generated with each guide RNA was confirmed by sequencing a fragment surrounding the target site (Fig. 3F, Supplementary Figure S3).

## Discussion

In this study we describe the development of a rapid, simple and efficient CRISPR-Cas9 system that can be used to edit or delete genes in any isolate of *C*. *parapsilosis*. The *CAS9* gene is expressed from a plasmid, which facilitates transformation. The plasmid is easily cured from the transformed strains, limiting expression of *CAS9*, and therefore reducing the likelihood of off-target effects. Our system allows the incorporation of specific mutations or deletions without the need to engineer auxotrophic strains, or to integrate and recycle *CAS9* or selectable markers.

We found that driving expression of guide RNAs from an *SNR52* promoter produced colonies of various sizes, from pinpricks to very large (Fig. 2B). The reason for this is unclear. *SNR52* is often used to drive expression of sgRNAs because it is unusual among pol III transcripts in that it has an upstream promoter^50^. We confirmed the location of the *C*. *parapsilosis SNR52* promoter using RNA-seq data^45^. However, we note that using *SNR52* to drive sgRNA expression is also relatively inefficient in *C*. *albicans* ^36^.

Surrounding the sgRNAs with ribozymes and expressing the construct from a pol II promoter greatly increased the efficiency of gene editing, up to 100% (Figs 2 and 3). A similar increase in efficiency was observed when pol II promoters were combined with CRISPR in *C*. *albicans* ^36^, and is likely to be related to increased intracellular levels of the guide RNAs. We found that efficiency varied with different target genes and different gRNAs. For example, two sgRNAs directed against *ADE2* had similar efficiencies in *C*. *parapsilosis* CLIB214 (Table 1). However only one of the two sgRNAs directed against *URA3* generated edited strains, and at a reduced efficiency compared to sgRNAs directed against *ADE2*.

We found that *ADE2* was edited even in the absence of repair template in some strains (Fig. 2, Table 1, Supplementary Fig. S3). Sequence analysis of adenine auxotrophs from four of these strains showed that this resulted from insertions or deletions at the predicted Cas9 cleavage site (Supplementary Fig. S3). In addition, even in the presence of a repair template, six adenine auxotrophs in *C*. *parapsilosis* 81/040 resulted from insertion of a single base at the Cas9 site, rather than incorporation of the repair template (Supplementary Fig. S3). These editing events most likely arise from repair by non-homologous end joining (NHEJ), which may be more efficient in some backgrounds. We found that for most strains, the transformation efficiency was lower in the absence of the repair template (e.g. Fig. 2E). In *C*. *albicans* it has been suggested that repairing toxic double-stranded breaks by NHEJ is rare, leading to reduced transformation efficiencies in the absence of a repair template^36^. It is possible that this is also true in *C*. *parapsilosis*, but that in some strains (such as 81/040) homology-directed repair is also low.

Our system is the first to apply CRISPR-Cas9 in *C*. *parapsilosis*, and it also has some advantages over some of the approaches described in *C*. *albicans*. For example, a plasmid-based system can be applied in any isolate, without the need for auxotrophies or additional dominant selectable markers. Everything needed (*CAS9*, sgRNA, repair template) is provided in a single one-step transformation, using one selectable marker, nourseothricin resistance. The genomes of edited and deleted strains are completely “scar-free” if necessary, with no extraneous sequences added, and only the target locus is disrupted.

In many of the *C*. *albicans* protocols, *CAS9* is either integrated in- and sometimes subsequently recycled from- the genome^33, 36, 37^, or multiple selectable markers are required^34, 35^. One difficulty with integrating *CAS9* is that it is impossible to control expression, and the level of off-target editing in *Candida* species is currently unknown. In mammalian CRISPR/Cas9 systems it has been shown that reducing the half life of Cas9 in the cells can reduce off-target effects^51, 52^. In the system described here, the plasmid encoding Cas9 is very quickly eliminated from *C*. *parapsilosis* by passaging without selection.

The fact that pSAT1 can be easily lost means that it is also possible to sequentially target many genes in any isolate of *C*. *parapsilosis*. Two of the recent CRISPR-based methods applied in *C*. *albicans* allow marker recycling, and therefore several targets can be edited^35, 37^. However, the recycling strategy of Huang *et al*.^35^ requires auxotrophic parental strains, with at least two selectable markers. Nguyen *et al*.^37^ use a recyclable nourseothricin resistance cassette integrated at *HIS1* or at *LEU2*. The HIS-FLP system facilitates gene editing in any *C*. *albicans* strain, but one allele of *HIS1* is interrupted by an FRT site following the first editing event^37^. The LEUpOUT system, which can be used for iterative marker-less editing in *C*. *albicans*, requires that one *LEU2* allele is disrupted in the parental strain. Using our system, we edited two genes (*URA3* and *ADE2*) in a single strain of *C*. *parapsilosis* by consecutive transformation with two plasmids expressing different sgRNAs (Fig. 3). The only requirement is that the plasmid containing the guide RNA is cured by passaging without selection before each step. In theory, gene editing can be carried out indefinitely without introducing any extraneous sequences in the genome. It may also be possible to generate multiple mutations in the same strain by co-transforming both plasmids at the same time. Nguyen *et al*.^37^ and Norton *et al*.^41^ showed that CRISPR can be used to target two genes simultaneously in *C*. *albicans* and *C*. *tropicalis* respectively, although the efficiency was low.

One of our goals was to develop a gene editing/deletion system that could easily be used with any target gene in any strain, and ideally could be applied to generating large numbers of gene knockouts. We have shown that our system is efficient even when applied to genes with no known phenotype (Fig. 3). We have also shown that our plasmid-based system works efficiently in almost all strains tested, providing a valuable tool for investigating the role of individual genes in multiple genetic backgrounds. For example, Pannanusorn *et al*.^53^ have shown that Bcr1, a regulator of biofilm development in *C*. *parapsilosis* ^25, 32, 54^ is important only in isolates that make relatively small quantities of biofilm.

We can use the repair template to incorporate unique tags into each mutant strain, facilitating downstream competition experiments^55^, or design the template to delete rather than edit genes (Fig. 3F). At present, the slowest step is replacing the target guide RNA in the pSAT1 plasmid. Because the first 6 bases of the hammerhead ribozyme form a stem with the beginning of the gRNA target, the entire region must be replaced. This is accomplished by primer extension from two oligonucleotides, generating a ~100 base pair fragment which is finally introduced into pSAT1 in a two-step process by Gibson assembly (Supplementary Methods). This approach may be simplified in future iterations of the method, perhaps by replacing the hammerhead ribozyme with a tRNA sequence^36^. It may also be possible to extend plasmid-based systems to other *Candida* species in the CUG clade. For example, a similar method has very recently been described in the CUG-clade species *Scheffersomyces stipitis* ^56^. The system we describe here is simple and remarkably efficient, with many potential applications in *C*. *parapsilosis*.

## Material and Methods

### Strains and media

All *C*. *parapsilosis* strains used in this study (Supplementary Table S2) were grown in YPD medium (1% yeast extract, 2% peptone, 2% glucose) or on YPD plates (YPD + 2% agar) at 30 °C. Transformants were selected on YPD agar supplemented with 200 μg/ml nourseothricin (Werner Bioagents Jena, Germany). Auxotrophies were confirmed by growing mutant strains on synthetic complete dropout media (0.19% yeast nitrogen base without amino acids and ammonium sulfate, 0.5% ammonium sulfate, 2% glucose, 0.075% amino acid dropout mix, 2% agar). All the plasmids used in this study (Supplementary Table S3) were propagated in *Escherichia coli* DH5α cells (NEB, UK) by growing cells in LB media without NaCl (Formedium) supplemented with 100 μg/ml Ampicillin (Sigma).

### Synthesis of *CAS9*

The *Streptococcus pyogenes CAS9* gene sequence was adapted by using the optimal *S*. *cerevisiae* codon for every amino acid throughout the gene, including TTG for Leu, and an NLS was added at the C terminus. This *sequence* was synthesized as six gBlocks that were combined by Gibson assembly (IDT, Supplementary Fig. S1, GenBank accession number MF421322). The 5′ ends of gBlock-1 and -4 and the 3′ ends of gBlocks-3 and -6 include 25–50 bp that overlap with the sequence surrounding EcoRV in plasmid pUC57 (GenScript). Each gBlock includes 25–50 bp overlap with the adjacent gBlock. gBlocks were amplified by PCR using primers A-L (Supplementary Table S1). gBlocks-1/2/3 and gBlocks-4/5/6 were independently cloned into EcoRV-digested pUC57 by Gibson assembly (NEB)^57^, generating plasmids pUC57-CAS9fr1 and pUC57-CAS9fr2. The inserts from each plasmid were amplified using primers CpCAS9-GA1 + 2 and CpCAS9-GA3 + 4 respectively. Primers CpCAS9-GA2 and CpCAS9-GA3 overlap with each other by 28 bases, and CpCAS9-GA1 and CpCAS9-GA3 overlap with EcoRV-cut pUC57 by 30–40 bases. The two fragments were cloned into pUC57 by Gibson assembly, generating pUC57-CAS9.

### Construction of pSAT1

679 bp upstream and 876 bp downstream of the *C*. *parapsilosis TEF1* gene were amplified from genomic DNA using primers TEF1p_AgeI_Fw + Rv and TEF1t_BamHI_Fw + Rv (Supplementary Table S1), which include AgeI or BamHI recognition sites respectively. The fragments were ligated into pUC57-CAS9 cut with the indicated enzymes, generating pUC57-pCAS9t. ARS7^43^ was amplified from the plasmid pGIZI using primers GA_CpARS_Fw and GA_CpARS_Rv that include 40 bp overlapping with AatII-digested pUC57-pCAS9t, and was cloned at this site by Gibson Assembly generating pUC57-ARS-pCAS9t. *SAT1*, conferring resistance to nourseothricin, was amplified from the plasmid pSFS2A^58^ using primers SapI_CaSAT1_Fw and SapI_CaSAT1_Rv that contain SapI recognition sites at the 5′ and 3′ ends. SAT1 was then ligated into SapI-digested pUC57-ARS-pCAS9t generating pSAT1. The sequence of the CAS9 insert was confirmed by Sanger sequencing (MWG/Eurofins) using the primers listed in Supplementary Table S1.

### Generation of pSNR and pRIBO plasmids

Guide RNAs *were* designed by using Eukaryotic Pathogen CRISPR guide RNA Design Tool^46^. Two synthetic constructs (Eurofins MWG), SNR-ADE2-B and GAPDH-HH-ADE2B-HDV, were designed to express the sgRNAB targeting *ADE2* from RNA pol III and RNA pol II promoters, respectively (Supplementary Figs S5, S6). The SNR-ADE2-B construct includes the *C*. *parapsilosis SNR52* promoter followed by a guide RNA targeting *ADE2* (guide B), the scaffold RNA sequence, and the *SUP4* terminator from *Saccharomyces cerevisiae* ^47^. In the GAPDH-HH-ADE2B-HDV construct, the guide B and the scaffold RNA are flanked by a 5′ hammerhead (HH) and a 3′ Hepatitis Delta virus (HDV) ribozymes^48^. Expression is driven by the *C*. *parapsilosis GAPDH* (*CPAR2_808670*) promoter and terminator. Each construct was cloned into SacI/BamHI digested pUC57 generating pUC57_CpSNR52p_ADE2_sgRNAB and pUC57_HH_HDV_ADE2_sgRNAB (Supplementary Table S3). New guide RNAs were introduced into pUC57_HH_HDV_ADE2_sgRNAB by replacing the HH-guide RNA segment by Gibson Assembly (New England Biolabs, UK). The HH-guide RNA inserts were generated by primer extension from two oligonucleotide primers overlapping at their 3′ ends (Supplementary Tables S1, S3). The cassettes were moved from the pUC57 background to pSAT1 by PCR amplification and Gibson Assembly into NruI digested pSAT1, generating pSNR and pRIBO plasmids (Supplementary Tables S1, S3).

### CRISPR-Cas9 mutagenesis in *C*. *parapsilosis*

*C*. *parapsilosis* strains were transformed^32^ with 5 μg of the relevant plasmid, alone or in combination with 5 μg of the corresponding repair template. Repair templates (80–108 bp) were generated with ExTaq DNA polymerase (TaKaRa Bio, USA) by primer extension from two oligonucleotide primers with 20 bp overlaps at the 3′-ends (Supplementary Table S1). Repair templates were designed to encode two consecutive stop codons and to mutate the PAM site, or to remove sequences between start and stop codon of the target gene. Barcodes were included where indicated (Supplementary Table S1). Nourseothricin-resistant transformants were patched onto SC lacking either adenine or uracil where indicated, and screened by allele-specific colony PCR. Representative mutants were sequenced by Sanger sequencing (MWG/Eurofins). Transformations of clinical isolates with pRIBO_ADE2-B were performed at least twice by two different people. Loss of pSAT1 constructs was induced by patching transformants onto YPD agar without selection and re-patching every 48 h until they no longer grew on parallel YPD agar plates containing 200 μg/mL nourseothricin. Resistance to nourseothricin was usually lost after just two passages without selection.

### *CAS9* expression

*C*. *parapsilosis* CLIB214 transformed with pSAT1 was cultured overnight in 5 mL YPD containing nourseothricin (100 μg/mL) and total RNA was extracted using the ISOLATE II RNA Mini Kit (Bioline, BIO-52072). cDNA was generated using MMLV reverse transcriptase with oligo dT primers (Promega). PCR was performed using primers C-CpCAS9 gBlock2-Fw and D-CpCAS9 gBlock2-Rv that amplify a 740 bp fragment within the *CAS9* coding sequence. Primers amplifying an internal sequence from *CpACT1* were used as a reference.

The full images of all gels are provided in the Supplementary Material.

### Data availability

There is no large-scale data associated with this manuscript. All constructs and strains are available on request.

## Electronic supplementary material

Supplementary Information

## References

[1] Pfaller MA (2010). Results from the ARTEMIS DISK Global Antifungal Surveillance Study, 1997 to 2007: a 10.5-year analysis of susceptibilities of *Candida* species to fluconazole and voriconazole as determined by CLSI standardized disk diffusion. J. Clin. Microbiol..

[2] Diekema DJ (2002). Epidemiology of candidemia: 3-year results from the emerging infections and the epidemiology of Iowa organisms study. J. Clin. Microbiol..

[3] Delfino D (2014). Potential association of specific *Candida parapsilosis* genotypes, bloodstream infections and colonization of health workers’ hands. Clin Microbiol Infect.

[4] van Asbeck EC, Huang YC, Markham AN, Clemons KV, Stevens DA (2007). *Candida parapsilosis* fungemia in neonates: genotyping results suggest healthcare workers hands as source, and review of published studies. Mycopathologia.

[5] Posteraro B (2004). *Candida parapsilosis* bloodstream infection in pediatric oncology patients: results of an epidemiologic investigation. Infect. Control Hosp. Epidemiol..

[6] Levin AS (1998). *Candida parapsilosis* fungemia associated with implantable and semi-implantable central venous catheters and the hands of healthcare workers. Diagn. Microbiol. Infect. Dis..

[7] Riley R (2016). Comparative genomics of biotechnologically important yeasts. Proc. Natl. Acad. Sci. USA.

[8] Santos MA, Tuite MF (1995). The CUG codon is decoded *in vivo* as serine and not leucine in *Candida albicans*. Nucleic Acids Res..

[9] Hittinger CT (2015). Genomics and the making of yeast biodiversity. Curr. Opin. Genet. Dev..

[10] Shen XX (2016). Reconstructing the backbone of the Saccharomycotina yeast phylogeny using genome-scale data. G3 (Bethesda).

[11] Jeffries TW (2007). Genome sequence of the lignocellulose-bioconverting and xylose-fermenting yeast *Pichia stipitis*. Nat. Biotechnol..

[12] Wohlbach DJ (2011). Comparative genomics of xylose-fermenting fungi for enhanced biofuel production. Proc. Natl. Acad. Sci. USA.

[13] Daniel HM, Lachance MA, Kurtzman CP (2014). On the reclassification of species assigned to *Candida* and other anamorphic ascomycetous yeast genera based on phylogenetic circumscription. Antonie Van Leeuwenhoek.

[14] James SA, Collins MD, Roberts IN (1994). The genetic relationship of *Lodderomyces elongisporus* to other ascomycete yeast species as revealed by small-subunit rRNA gene sequences. Lett. Appl. Microbiol..

[15] Bennett RJ, Johnson AD (2005). Mating in *Candida albicans* and the search for a sexual cycle. Annu. Rev. Microbiol..

[16] Porman AM, Alby K, Hirakawa MP, Bennett RJ (2011). Discovery of a phenotypic switch regulating sexual mating in the opportunistic fungal pathogen *Candida tropicalis*. Proc. Natl. Acad. Sci. USA.

[17] Pujol C (2004). The closely related species *Candida albicans* and *Candida dubliniensis* can mate. Eukaryot. Cell.

[18] Forche A (2008). The parasexual cycle in *Candida albicans* provides an alternative pathway to meiosis for the formation of recombinant strains. PLoS Biol.

[19] Butler G (2009). Evolution of pathogenicity and sexual reproduction in eight *Candida* genomes. Nature.

[20] Sai S, Holland L, McGee CF, Lynch DB, Butler G (2011). Evolution of mating within the *Candida parapsilosis* species group. Eukaryot. Cell.

[21] Pryszcz LP, Nemeth T, Gacser A, Gabaldon T (2013). Unexpected genomic variability in clinical and environmental strains of the pathogenic yeast *Candida parapsilosis*. Genome Biol Evol.

[22] Papon N (2012). Deus ex *Candida* genetics: overcoming the hurdles for the development of a molecular toolbox in the CTG clade. Microbiology.

[23] Roemer T (2003). Large-scale essential gene identification in *Candida albicans* and applications to antifungal drug discovery. Mol. Microbiol..

[24] Reuss O, Vik A, Kolter R, Morschhauser J (2004). The *SAT1* flipper, an optimized tool for gene disruption in *Candida albicans*. Gene.

[25] Ding C, Butler G (2007). Development of a gene knockout system in *Candida parapsilosis* reveals a conserved role for *BCR1* in biofilm formation. Eukaryot. Cell.

[26] Gacser A, Trofa D, Schafer W, Nosanchuk JD (2007). Targeted gene deletion in *Candida parapsilosis* demonstrates the role of secreted lipase in virulence. J. Clin. Invest..

[27] Bertini A (2016). Targeted gene disruption in *Candida parapsilosis* demonstrates a role for CPAR2_404800 in adhesion to a biotic surface and in a murine model of ascending urinary tract infection. Virulence.

[28] Millerioux Y (2011). Drug-resistant cassettes for the efficient transformation of Candida guilliermondii wild-type strains. FEMS Yeast Res.

[29] Dunkel N (2013). Roles of different peptide transporters in nutrient acquisition in Candida albicans. Eukaryot. Cell.

[30] Noble SM, Johnson AD (2005). Strains and strategies for large-scale gene deletion studies of the diploid human fungal pathogen *Candida albicans*. Eukaryot. Cell.

[31] Noble SM, French S, Kohn LA, Chen V, Johnson AD (2010). Systematic screens of a *Candida albicans* homozygous deletion library decouple morphogenetic switching and pathogenicity. Nat. Genet..

[32] Holland LM (2014). Comparative phenotypic analysis of the major fungal pathogens Candida parapsilosis and Candida albicans. PLoS Pathog.

[33] Vyas VK, Barrasa MI, Fink GR (2015). A CRISPR system permits genetic engineering of essential genes and gene families. Sci Adv.

[34] Min K, Ichikawa Y, Woolford CA, Mitchell AP (2016). *Candida albicans* gene deletion with a transient CRISPR-Cas9 system. mSphere.

[35] Huang MY, Mitchell AP (2017). Marker recycling in *Candida albicans* through CRISPR-Cas9-Induced Marker Excision. mSphere.

[36] Ng H, Dean N (2017). Dramatic Improvement of CRISPR/Cas9 Editing in *Candida albicans* by Increased Single Guide RNA Expression. mSphere.

[37] Nguyen N, Quail MF, Hernday AD (2017). An efficient, rapid and recyclable sytem for CRISPR-mediated genome editing in *Candida albicans*. mSphere.

[38] Mojica FJ, Diez-Villasenor C, Garcia-Martinez J, Soria E (2005). Intervening sequences of regularly spaced prokaryotic repeats derive from foreign genetic elements. J. Mol. Evol..

[39] Jinek M (2012). A programmable dual-RNA-guided DNA endonuclease in adaptive bacterial immunity. Science.

[40] Sternberg SH, Doudna JA (2015). Expanding the Biologist’s Toolkit with CRISPR-Cas9. Mol. Cell.

[41] Norton EL, Sherwood RK, Bennett RJ (2017). Development of a CRISPR-Cas9 sytem for efficient genome editing of Candida lusitaniae. mSphere.

[42] Grahl N, Demers EG, Crocker AW, Hogan DA (2017). Use of RNA-protein complexes for genome editing in non-albicans *Candida* species. mSphere.

[43] Nosek J (2002). Genetic manipulation of the pathogenic yeast *Candida parapsilosis*. Curr. Genet..

[44] Nodvig CS, Nielsen JB, Kogle ME, Mortensen UH (2015). A CRISPR-Cas9 System for genetic engineering of filamentous fungi. PLoS One.

[45] Donovan PD, Schroder MS, Higgins DG, Butler G (2016). Identification of Non-coding RNAs in the *Candida parapsilosis* species group. PLoS One.

[46] Peng D, Tarleton R (2015). EuPaGDT: a web tool tailored to design CRISPR guide RNAs for eukaryotic pathogens. Microb Genom.

[47] DiCarlo JE (2013). Genome engineering in *Saccharomyces cerevisiae* using CRISPR-Cas systems. Nucleic Acids Res..

[48] Gao Y, Zhao Y (2014). Self-processing of ribozyme-flanked RNAs into guide RNAs *in vitro* and *in vivo* for CRISPR-mediated genome editing. J Integr Plant Biol.

[49] Jacobs JZ, Ciccaglione KM, Tournier V, Zaratiegui M (2014). Implementation of the CRISPR-Cas9 system in fission yeast. Nat Commun.

[50] Marck C (2006). The RNA polymerase III-dependent family of genes in hemiascomycetes: comparative RNomics, decoding strategies, transcription and evolutionary implications. Nucleic Acids Res..

[51] Kim S, Kim D, Cho SW, Kim J, Kim JS (2014). Highly efficient RNA-guided genome editing in human cells via delivery of purified Cas9 ribonucleoproteins. Genome Res..

[52] Koo T, Lee J, Kim JS (2015). Measuring and reducing Off-Target activities of programmable nucleases including CRISPR-Cas9. Mol. Cells.

[53] Pannanusorn S (2014). Characterization of biofilm formation and the role of *BCR1* in clinical isolates of *Candida parapsilosis*. Eukaryot Cell.

[54] Ding C (2011). Conserved and divergent roles of Bcr1 and CFEM proteins in *Candida parapsilosis* and *Candida albicans*. PLoS One.

[55] Pande K, Chen C, Noble SM (2013). Passage through the mammalian gut triggers a phenotypic switch that promotes *Candida albicans* commensalism. Nat. Genet..

[56] Cao, M. *et al*. Centromeric DNA facilitates nonconventional yeast genetic engineering. *ACS Synth Biol*, in press, doi:10.1021/acssynbio.7b00046 (2017). 10.1021/acssynbio.7b00046 28391682

[57] Gibson DG (2009). Enzymatic assembly of DNA molecules up to several hundred kilobases. Nature Methods.

[58] Morschhauser J, Michel S, Staib P (1999). Sequential gene disruption in *Candida albicans* by FLP-mediated site-specific recombination. Mol. Microbiol..

