# Supplementary material for: Gene editing in clinical isolates of Candida parapsilosis using CRISPR/Cas9
Source: Sci Rep. 2017 Aug 14;7:8051. doi: 10.1038/s41598-017-08500-1 (PMC5556056; doi:10.1038/s41598-017-08500-1)
Supplement: Supplementary file 1 — Supplementary Information [file 41598_2017_8500_MOESM1_ESM.pdf]

Supplementary Materials for

Gene editing in clinical isolates of *Candida parapsilosis* using CRISPR/Cas9

Lisa Lombardi[1], Siobhán A. Turner[1], Fang Zhao and Geraldine Butler*
School of Biomolecular and Biomedical Science, Conway Institute, University College Dublin, Belfield, Dublin 4, Ireland.

[1]These authors made equal contributions.

*Corresponding author: Geraldine Butler, School of Biomolecular and Biomedical Science, Conway Institute, University College Dublin, Belfield, Dublin 4, Ireland

This file includes:

Fig. S1: Codon-optimized Cas9 page 2
Fig. S2: pSNR and pRIBO plasmids rarely integrate into the genome page 4
Fig. S3: Sequence of edited genes page 5
Fig. S4: Generation of *ura3* and *ura3/ade2* double mutants page 7
Fig. S5: Sequence of synthetic construct SNR-ADE2B page 8
Fig. S6: Sequence of synthetic construct HH-ADE2B-HDV page 8
Figs. 1-3: Complete gels from Fig. 1B, Fig. 2C and Fig. 3D and 3E. page 9
Table S1: Oligonucleotide sequences used in this study page 11
Table S2: Strains used in this study page 17
Table S3: Plasmids generated in this study page 20
Supplementary methods: Instructions for using CRISPR-Cas9 in *C. parapsilosis* page 22

Fig. S1. Codon Optimized Cas9. The Nuclear Localization Sequence is indicated in red.

```
   1 ATGGACAAGAAGTAC TCTATCGGTTTGGAC ATCGGTACTAACTCT GTTGGTTGGGCTGTT ATCACTGACGAATAC
   1  M  D  K  K  Y   S  I  G  L  D   I  G  T  N  S   V  G  W  A  V   I  T  D  E  Y
  76 AAGGTTCCATCTAAG AAGTTCAAGGTTTTG GGTAACACTGACAGA CACTCTATCAAGAAG AACTTGATCGGTGCT
  26  K  V  P  S  K   K  F  K  V  L   G  N  T  D  R   H  S  I  K  K   N  L  I  G  A
 151 TTGTTGTTCGACTCT GGTGAAACTGCTGAA GCTACTAGATTGAAG AGAACTGCTAGAAGA AGATACACTAGAAGA
  51  L  L  F  D  S   G  E  T  A  E   A  T  R  L  K   R  T  A  R  R   R  Y  T  R  R
 226 AAGAACAGAATCTGT TACTTGCAAGAAATC TTCTCTAACGAAATG GCTAAGGTTGACGAC TCTTTCTTCCACAGA
  76  K  N  R  I  C   Y  L  Q  E  I   F  S  N  E  M   A  K  V  D  D   S  F  F  H  R
 301 TTGGAAGAATCTTTC TTGGTTGAAGAAGAC AAGAAGCACGAAAGA CACCCAATCTTCGGT AACATCGTTGACGAA
 101  L  E  E  S  F   L  V  E  E  D   K  K  H  E  R   H  P  I  F  G   N  I  V  D  E
 376 GTTGCTTACCACGAA AAGTACCCAACTATC TACCACTTGAGAAAG AAGTTGGTTGACTCT ACTGACAAGGCTGAC
 126  V  A  Y  H  E   K  Y  P  T  I   Y  H  L  R  K   K  L  V  D  S   T  D  K  A  D
 451 TTGAGATTGATCTAC TTGGCTTTGGCTCAC ATGATCAAGTTCAGA GGTCACTTCTTGATC GAAGGTGACTTGAAC
 151  L  R  L  I  Y   L  A  L  A  H   M  I  K  F  R   G  H  F  L  I   E  G  D  L  N
 526 CCAGACAACTCTGAC GTTGACAAGTTGTTC ATCCAATTGGTTCAA ACTTACAACCAATTG TTCGAAGAAAACCCA
 176  P  D  N  S  D   V  D  K  L  F   I  Q  L  V  Q   T  Y  N  Q  L   F  E  E  N  P
 601 ATCAACGCTTCTGGT GTTGACGCTAAGGCT ATCTTGTCTGCTAGA TTGTCTAAGTCTAGA AGATTGGAAAACTTG
 201  I  N  A  S  G   V  D  A  K  A   I  L  S  A  R   L  S  K  S  R   R  L  E  N  L
 676 ATCGCTCAATTGCCA GGTGAAAAGAAGAAC GGTTTGTTCGGTAAC TTGATCGCTTTGTCT TTGGGTTTGACTCCA
 226  I  A  Q  L  P   G  E  K  K  N   G  L  F  G  N   L  I  A  L  S   L  G  L  T  P
 751 AACTTCAAGTCTAAC TTCGACTTGGCTGAA GACGCTAAGTTGCAA TTGTCTAAGGACACT TACGACGACGACTTG
 251  N  F  K  S  N   F  D  L  A  E   D  A  K  L  Q   L  S  K  D  T   Y  D  D  D  L
 826 GACAACTTGTTGGCT CAAATCGGTGACCAA TACGCTGACTTGTTC TTGGCTGCTAAGAAC TTGTCTGACGCTATC
 276  D  N  L  L  A   Q  I  G  D  Q   Y  A  D  L  F   L  A  A  K  N   L  S  D  A  I
 901 TTGTTGTCTGACATC TTGAGAGTTAACACT GAAATCACTAAGGCT CCATTGTCTGCTTCT ATGATCAAGAGATAC
 301  L  L  S  D  I   L  R  V  N  T   E  I  T  K  A   P  L  S  A  S   M  I  K  R  Y
 976 GACGAACACCACCAA GACTTGACTTTGTTG AAGGCTTTGGTTAGA CAACAATTGCCAGAA AAGTACAAGGAAATC
 326  D  E  H  H  Q   D  L  T  L  L   K  A  L  V  R   Q  Q  L  P  E   K  Y  K  E  I
1051 TTCTTCGACCAATCT AAGAACGGTTACGCT GGTTACATCGACGGT GGTGCTTCTCAAGAA GAATTCTACAAGTTC
 351  F  F  D  Q  S   K  N  G  Y  A   G  Y  I  D  G   G  A  S  Q  E   E  F  Y  K  F
1126 ATCAAGCCAATCTTG GAAAAGATGGACGGT ACTGAAGAATTGTTG GTTAAGTTGAACAGA GAAGACTTGTTGAGA
 376  I  K  P  I  L   E  K  M  D  G   T  E  E  L  L   V  K  L  N  R   E  D  L  L  R
1201 AAGCAAAGAACTTTC GACAACGGTTCTATC CCACACCAAATCCAC TTGGGTGAATTGCAC GCTATCTTGAGAAGA
 401  K  Q  R  T  F   D  N  G  S  I   P  H  Q  I  H   L  G  E  L  H   A  I  L  R  R
1276 CAAGAAGACTTCTAC CCATTCTTGAAGGAC AACAGAGAAAAGATC GAAAAGATCTTGACT TTCAGAATCCCATAC
 426  Q  E  D  F  Y   P  F  L  K  D   N  R  E  K  I   E  K  I  L  T   F  R  I  P  Y
1351 TACGTTGGTCCATTG GCTAGAGGTAACTCT AGATTCGCTTGGATG ACTAGAAAGTCTGAA GAAACTATCACTCCA
 451  Y  V  G  P  L   A  R  G  N  S   R  F  A  W  M   T  R  K  S  E   E  T  I  T  P
1426 TGGAACTTCGAAGAA GTTGTTGACAAGGGT GCTTCTGCTCAATCT TTCATCGAAAGAATG ACTAACTTCGACAAG
 476  W  N  F  E  E   V  V  D  K  G   A  S  A  Q  S   F  I  E  R  M   T  N  F  D  K
1501 AACTTGCCAAACGAA AAGGTTTTGCCAAAG CACTCTTTGTTGTAC GAATACTTCACTGTT TACAACGAATTGACT
 501  N  L  P  N  E   K  V  L  P  K   H  S  L  L  Y   E  Y  F  T  V   Y  N  E  L  T
1576 AAGGTTAAGTACGTT ACTGAAGGTATGAGA AAGCCAGCTTTCTTG TCTGGTGAACAAAAG AAGGCTATCGTTGAC
 526  K  V  K  Y  V   T  E  G  M  R   K  P  A  F  L   S  G  E  Q  K   K  A  I  V  D
1651 TTGTTGTTCAAGACT AACAGAAAGGTTACT GTTAAGCAATTGAAG GAAGACTACTTCAAG AAGATCGAATGTTTC
 551  L  L  F  K  T   N  R  K  V  T   V  K  Q  L  K   E  D  Y  F  K   K  I  E  C  F
1726 GACTCTGTTGAAATC TCTGGTGTTGAAGAC AGATTCAACGCTTCT TTGGGTACTTACCAC GACTTGTTGAAGATC
 576  D  S  V  E  I   S  G  V  E  D   R  F  N  A  S   L  G  T  Y  H   D  L  L  K  I
1801 ATCAAGGACAAGGAC TTCTTGGACAACGAA GAAAACGAAGACATC TTGGAAGACATCGTT TTGACTTTGACTTTG
 601  I  K  D  K  D   F  L  D  N  E   E  N  E  D  I   L  E  D  I  V   L  T  L  T  L
1876 TTCGAAGACAGAGAA ATGATCGAAGAAAGA TTGAAGACTTACGCT CACTTGTTCGACGAC AAGGTTATGAAGCAA
 626  F  E  D  R  E   M  I  E  E  R   L  K  T  Y  A   H  L  F  D  D   K  V  M  K  Q
1951 TTGAAGAGAAGAAGA TACACTGGTTGGGGT AGATTGTCTAGAAAG TTGATCAACGGTATC AGAGACAAGCAATCT
 651  L  K  R  R  R   Y  T  G  W  G   R  L  S  R  K   L  I  N  G  I   R  D  K  Q  S
2026 GGTAAGACTATCTTG GACTTCTTGAAGTCT GACGGTTTCGCTAAC AGAAACTTCATGCAA TTGATCCACGACGAC
 676  G  K  T  I  L   D  F  L  K  S   D  G  F  A  N   R  N  F  M  Q   L  I  H  D  D
2101 TCTTTGACTTTCAAG GAAGACATCCAAAAG GCTCAAGTTTCTGGT CAAGGTGACTCTTTG CACGAACACATCGCT
 701  S  L  T  F  K   E  D  I  Q  K   A  Q  V  S  G   Q  G  D  S  L   H  E  H  I  A
2176 AACTTGGCTGGTTCT CCAGCTATCAAGAAG GGTATCTTGCAAACT GTTAAGGTTGTTGAC GAATTGGTTAAGGTT
 726  N  L  A  G  S   P  A  I  K  K   G  I  L  Q  T   V  K  V  V  D   E  L  V  K  V
2251 ATGGGTAGACACAAG CCAGAAAACATCGTT ATCGAAATGGCTAGA GAAAACCAAACTACT CAAAAGGGTCAAAAG
 751  M  G  R  H  K   P  E  N  I  V   I  E  M  A  R   E  N  Q  T  T   Q  K  G  Q  K
```

```
2326 AACTCTAGAGAAAGA ATGAAGAGAATCGAA GAAGGTATCAAGGAA TTGGGTTCTCAAATC TTGAAGGAACACCCA
 776  N  S  R  E  R   M  K  R  I  E   E  G  I  K  E   L  G  S  Q  I   L  K  E  H  P
2401 GTTGAAAACACTCAA TTGCAAAACGAAAAG TTGTACTTGTACTAC TTGCAAAACGGTAGA GACATGTACGTTGAC
 801  V  E  N  T  Q   L  Q  N  E  K   L  Y  L  Y  Y   L  Q  N  G  R   D  M  Y  V  D
2476 CAAGAATTGGACATC AACAGATTGTCTGAC TACGACGTTGACCAC ATCGTTCCACAATCT TTCTTGAAGGACGAC
 826  Q  E  L  D  I   N  R  L  S  D   Y  D  V  D  H   I  V  P  Q  S   F  L  K  D  D
2551 TCTATCGACAACAAG GTTTTGACTAGATCT GACAAGAACAGAGGT AAGTCTGACAACGTT CCATCTGAAGAAGTT
 851  S  I  D  N  K   V  L  T  R  S   D  K  N  R  G   K  S  D  N  V   P  S  E  E  V
2626 GTTAAGAAGATGAAG AACTACTGGAGACAA TTGTTGAACGCTAAG TTGATCACTCAAAGA AAGTTCGACAACTTG
 876  V  K  K  M  K   N  Y  W  R  Q   L  L  N  A  K   L  I  T  Q  R   K  F  D  N  L
2701 ACTAAGGCTGAAAGA GGTGGTTTGTCTGAA TTGGACAAGGCTGGT TTCATCAAGAGACAA TTGGTTGAAACTAGA
 901  T  K  A  E  R   G  G  L  S  E   L  D  K  A  G   F  I  K  R  Q   L  V  E  T  R
2776 CAAATCACTAAGCAC GTTGCTCAAATCTTG GACTCTAGAATGAAC ACTAAGTACGACGAA AACGACAAGTTGATC
 926  Q  I  T  K  H   V  A  Q  I  L   D  S  R  M  N   T  K  Y  D  E   N  D  K  L  I
2851 AGAGAAGTTAAGGTT ATCACTTTGAAGTCT AAGTTGGTTTCTGAC TTCAGAAAGGACTTC CAATTCTACAAGGTT
 951  R  E  V  K  V   I  T  L  K  S   K  L  V  S  D   F  R  K  D  F   Q  F  Y  K  V
2926 AGAGAAATCAACAAC TACCACCACGCTCAC GACGCTTACTTGAAC GCTGTTGTTGGTACT GCTTTGATCAAGAAG
 976  R  E  I  N  N   Y  H  H  A  H   D  A  Y  L  N   A  V  V  G  T   A  L  I  K  K
3001 TACCCAAAGTTGGAA TCTGAATTCGTTTAC GGTGACTACAAGGTT TACGACGTTAGAAAG ATGATCGCTAAGTCT
1001  Y  P  K  L  E   S  E  F  V  Y   G  D  Y  K  V   Y  D  V  R  K   M  I  A  K  S
3076 GAACAAGAAATCGGT AAGGCTACTGCTAAG TACTTCTTCTACTCT AACATCATGAACTTC TTCAAGACTGAAATC
1026  E  Q  E  I  G   K  A  T  A  K   Y  F  F  Y  S   N  I  M  N  F   F  K  T  E  I
3151 ACTTTGGCTAACGGT GAAATCAGAAAGAGA CCATTGATCGAAACT AACGGTGAAACTGGT GAAATCGTTTGGGAC
1051  T  L  A  N  G   E  I  R  K  R   P  L  I  E  T   N  G  E  T  G   E  I  V  W  D
3226 AAGGGTAGAGACTTC GCTACTGTTAGAAAG GTTTTGTCTATGCCA CAAGTTAACATCGTT AAGAAGACTGAAGTT
1076  K  G  R  D  F   A  T  V  R  K   V  L  S  M  P   Q  V  N  I  V   K  K  T  E  V
3301 CAAACTGGTGGTTTC TCTAAGGAATCTATC TTGCCAAAGAGAAAC TCTGACAAGTTGATC GCTAGAAAGAAGGAC
1101  Q  T  G  G  F   S  K  E  S  I   L  P  K  R  N   S  D  K  L  I   A  R  K  K  D
3376 TGGGACCCAAAGAAG TACGGTGGTTTCGAC TCTCCAACTGTTGCT TACTCTGTTTTGGTT GTTGCTAAGGTTGAA
1126  W  D  P  K  K   Y  G  G  F  D   S  P  T  V  A   Y  S  V  L  V   V  A  K  V  E
3451 AAGGGTAAGTCTAAG AAGTTGAAGTCTGTT AAGGAATTGTTGGGT ATCACTATCATGGAA AGATCTTCTTTCGAA
1151  K  G  K  S  K   K  L  K  S  V   K  E  L  L  G   I  T  I  M  E   R  S  S  F  E
3526 AAGAACCCAATCGAC TTCTTGGAAGCTAAG GGTTACAAGGAAGTT AAGAAGGACTTGATC ATCAAGTTGCCAAAG
1176  K  N  P  I  D   F  L  E  A  K   G  Y  K  E  V   K  K  D  L  I   I  K  L  P  K
3601 TACTCTTTGTTCGAA TTGGAAAACGGTAGA AAGAGAATGTTGGCT TCTGCTGGTGAATTG CAAAAGGGTAACGAA
1201  Y  S  L  F  E   L  E  N  G  R   K  R  M  L  A   S  A  G  E  L   Q  K  G  N  E
3676 TTGGCTTTGCCATCT AAGTACGTTAACTTC TTGTACTTGGCTTCT CACTACGAAAAGTTG AAGGGTTCTCCAGAA
1226  L  A  L  P  S   K  Y  V  N  F   L  Y  L  A  S   H  Y  E  K  L   K  G  S  P  E
3751 GACAACGAACAAAAG CAATTGTTCGTTGAA CAACACAAGCACTAC TTGGACGAAATCATC GAACAAATCTCTGAA
1251  D  N  E  Q  K   Q  L  F  V  E   Q  H  K  H  Y   L  D  E  I  I   E  Q  I  S  E
3826 TTCTCTAAGAGAGTT ATCTTGGCTGACGCT AACTTGGACAAGGTT TTGTCTGCTTACAAC AAGCACAGAGACAAG
1276  F  S  K  R  V   I  L  A  D  A   N  L  D  K  V   L  S  A  Y  N   K  H  R  D  K
3901 CCAATCAGAGAACAA GCTGAAAACATCATC CACTTGTTCACTTTG ACTAACTTGGGTGCT CCAGCTGCTTTCAAG
1301  P  I  R  E  Q   A  E  N  I  I   H  L  F  T  L   T  N  L  G  A   P  A  A  F  K
3976 TACTTCGACACTACT ATCGACAGAAAGAGA TACACTTCTACTAAG GAAGTTTTGGACGCT ACTTTGATCCACCAA
1326  Y  F  D  T  T   I  D  R  K  R   Y  T  S  T  K   E  V  L  D  A   T  L  I  H  Q
4051 TCTATCACTGGTTTG TACGAAACTAGAATC GACTTGTCTCAATTG GGTGGTGACTCTAGA GCTGACCCAAAGAAG
1351  S  I  T  G  L   Y  E  T  R  I   D  L  S  Q  L   G  G  D  S  R   A  D  P  K  K
4126 AAGAGAAAGGTTTAA
1376  K  R  K  V  *
```

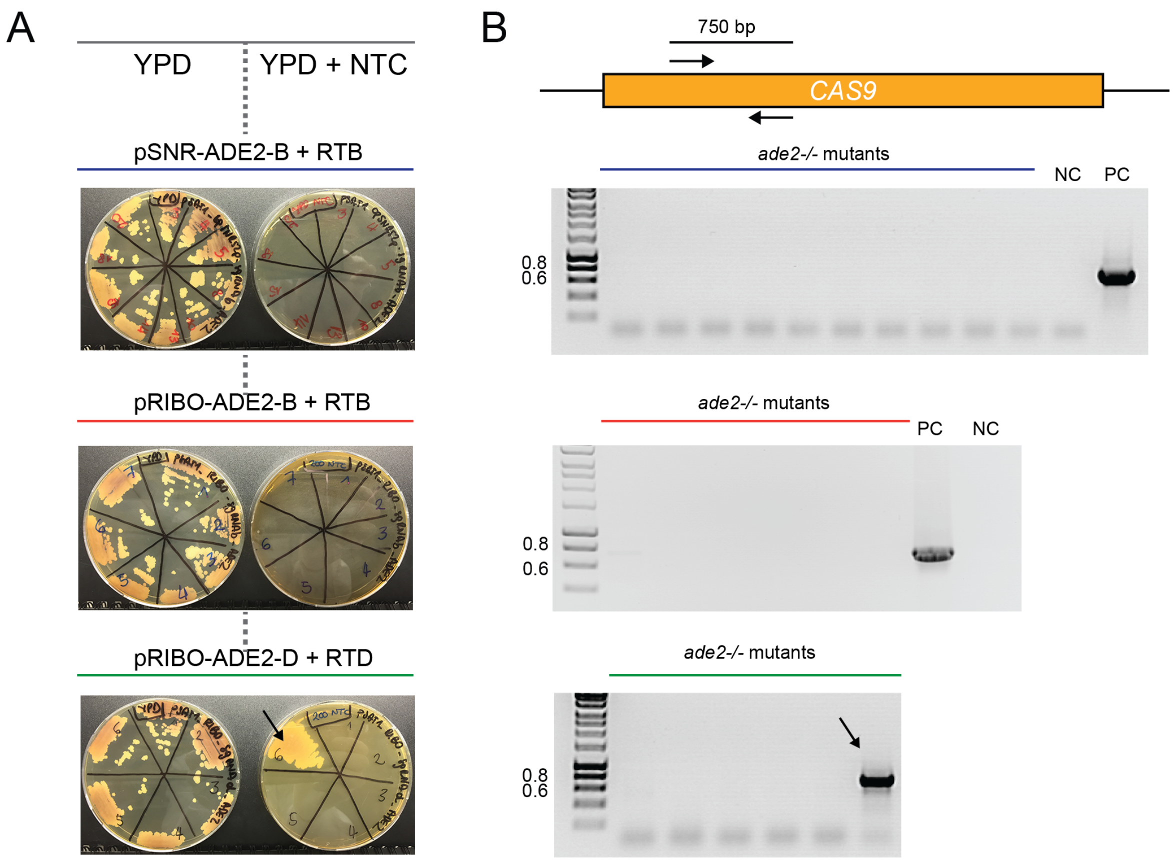

Figure S2: pSNR and pRIBO plasmids rarely integrate into the genome.

(A) Representative *ade2* disruptants generated by transforming *C. parapsilosis* CLIB214 with pSNR-ADE2-B or pRIBO-ADE2-B/D with the corresponding repair templates (RT) were streaked on YPD for 3 passages. Most of the mutant strains tested lost the ability to grow on selective media YPD + nourseothricin (NTC), with the exception of one colony, which is light pink (black arrow). (B) To test for integration of the plasmid, colony PCR amplifying a 750 bp fragment of *CAS9* sequence was performed using primers CpCAS9A_FWD and CpCAS9_REV (Table S1). A band was detected only in the NTC resistant colony (black arrow), suggesting that in this case pRIBO-ADE2-D may be integrated in the genome. NC = no DNA; PC = pSAT1 plasmid used as positive control.

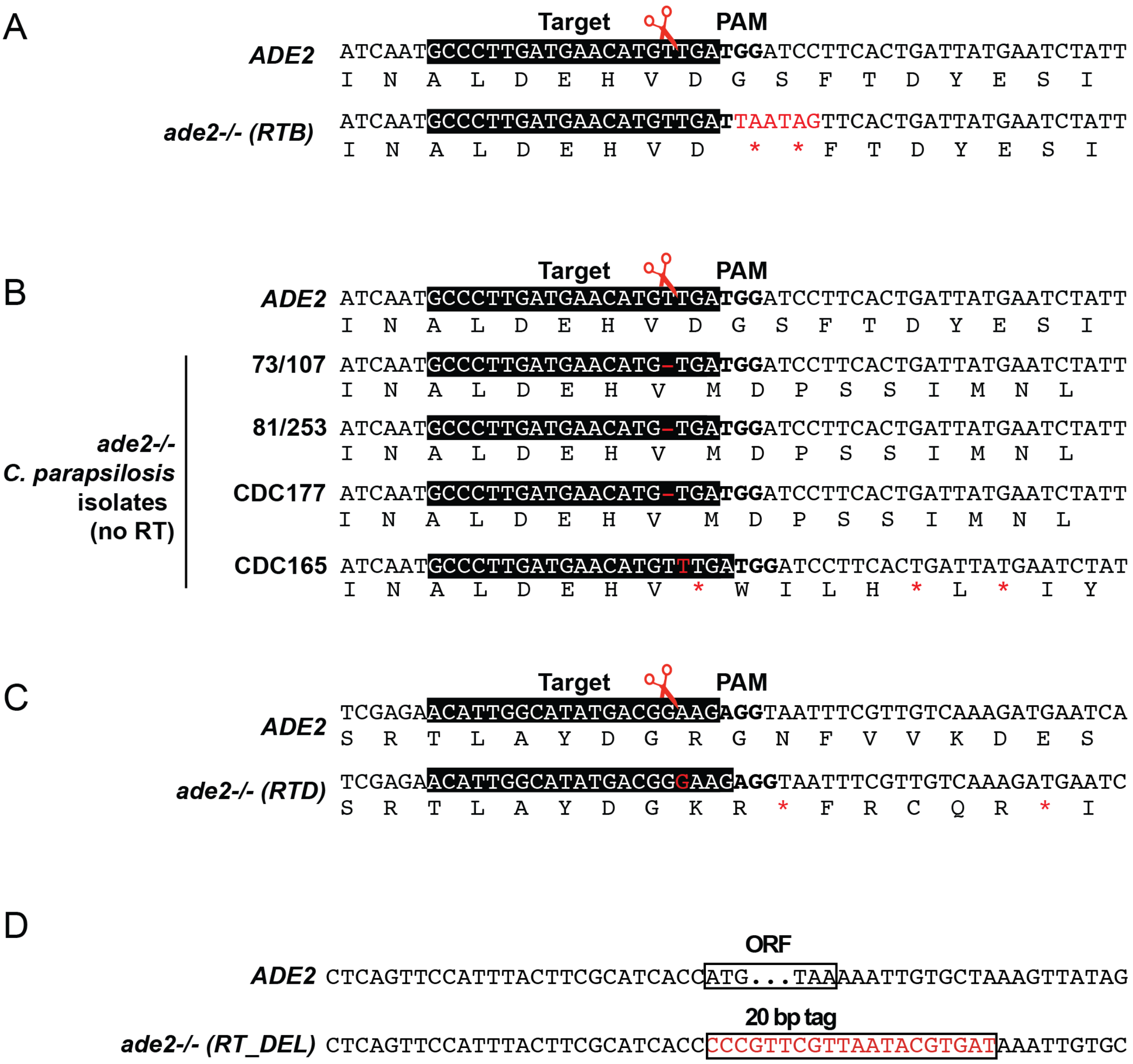

Fig. S3: Sequence of edited *ADE2* loci in multiple isolates.

(A) Representative *ade2* disruptants generated in *C. parapsilosis* CLIB214 and eight other clinical isolates (02-203, 103, 73/107, J960587, 81/042, 81/253, CDC165 and CDC177) using pRIBO-ADE2-B and repair template (RTB) were sequenced to confirm incorporation of the expected mutations. (B) Edited genes from four of these strains, generated in the absence of repair template, were sequenced to identify the causative mutation. Deletions or insertion of bases close to the Cas9 cleavage site upstream of the PAM sequence were identified. (C) One isolate, *C. parapsilosis* 81/040, yielded no adenine auxotrophs when transformed with pRIBO-ADE2-B. Using a different guide, pRIBO-ADE2-D pink transformants were produced at similar efficiency with and without the repair template (Fig. 2E). Many of these transformants were a mixture of pink and white cells. Sequencing of six

pink *C. parapsilosis* 81/040 colonies, obtained in the presence of the RTD, revealed an insertion of a single G adjacent to the Cas9 cleavage site. The repair template was not incorporated. (D) Sequencing of adenine auxotrophs obtained by transformation of *C. parapsilosis* 90-137 with pRIBO-ADE2-B or RIBO-ADE2-D and RT_DEL confirmed that the *ADE2* open reading frame (ORF) was replaced with a 20 bp unique tag. In (A) to (C) the scissors symbols show the expected Cas9 cleavage sites, the guide RNA target regions kfare highlighted with a black box, and the edited sequences are shown in red.

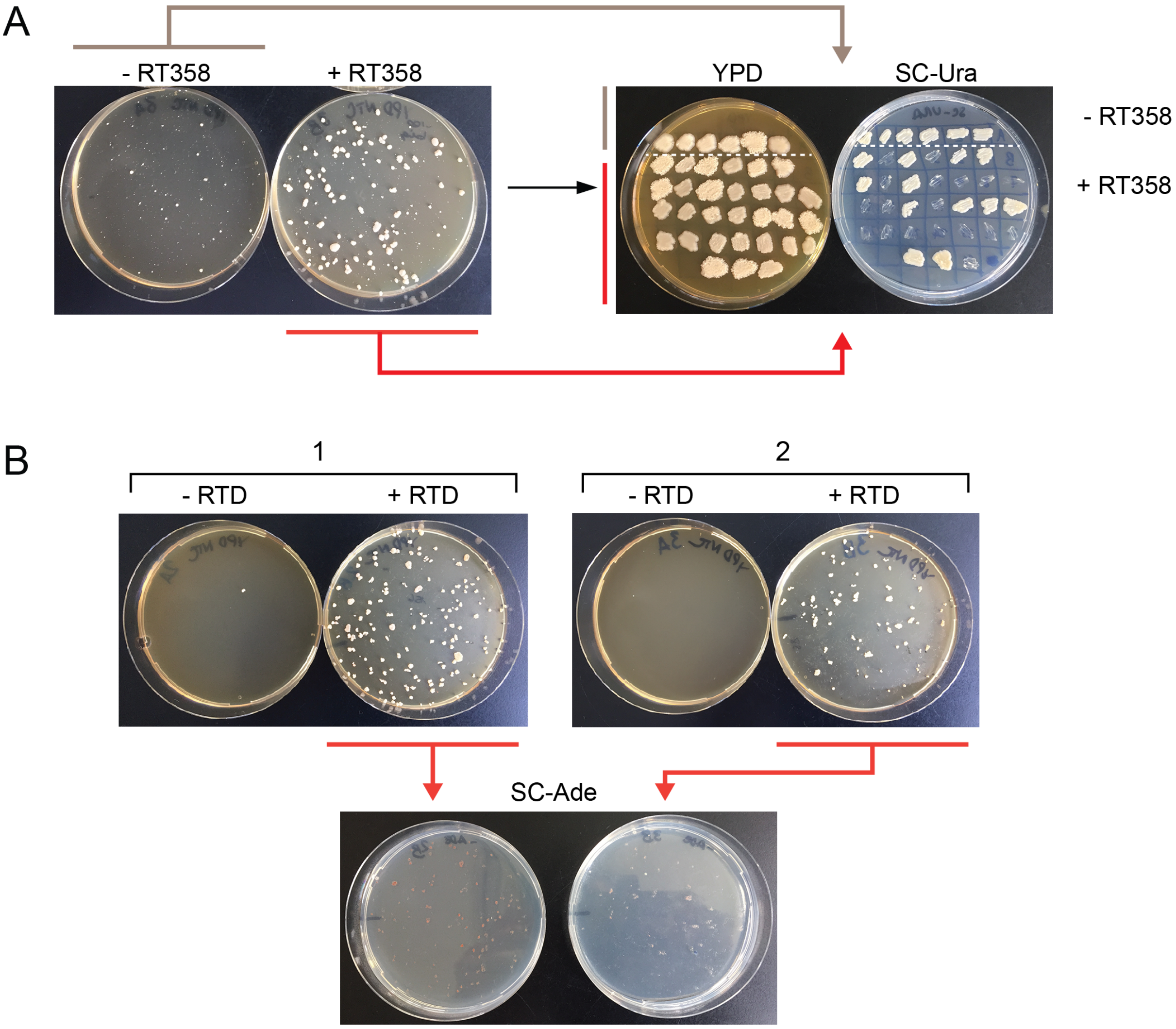

Figure S4: Generation of *ura3* and *ura3/ade2* double mutants

(A) Transformation of *C. parapsilosis* CLIB214 with pRIBO-URA3-358 with and without the repair template 358 (RT358). Representative nourseothricin (NTC) resistant colonies, 6 transformed only with the plasmid (brown arrow), and 30 transformed with both the plasmid and RT358 (red arrow), were patched on YPD and Sc-Ura. In the absence of RT358 no uracil auxotrophs were identified. In the presence of the RT358, 18/30 colonies were unable to grow in the absence of uracil. (B) One uracil auxotroph (CRISPR_LL5), which was confirmed by sequencing, was used as the parental strain to generate the double *ura3/ade2* mutant. Two independent transformations (1 and 2) were carried out. Cells were transformed with pRIBO-ADE2-D in the presence or absence of repair template D (RTD). In both independent experiments all the NTC resistant transformants (+RTD) failed to grow after replica plating on Sc-Ade.

Fig. S5: Sequence of SNR-ADE2B

GAGCTCTTAAGATATAGTTATCGGTTTTAATTCTAGTTTAATACAAAGGGTTGGATTTGTTAAGTC
GACAGTTCTTGGATGATATGTGATAATGTAGTAAAATATTTATATCAAATTGATACAAAATATCAAG
TTCAGAGAAGAGCTGAAAACATTAAGCCAAACAATAATGAAAACCTCCTCATCACAAGAAGACAA
TCAAAATGCATTGTTTATGCAAATGCAAAGTGCCATCGGTCAGATGCAGTTCAAAGTTTAAACAAC
CAAATTCACGTGTTTGAAAGTTCAGAATCCCTTCCCTATATAAACTAATTGAAAAATTTTGGACCTC
GTTGGCGTATTGGACTATGATAACGACATCCATTTTGGAGAACGTATGTGGCTCTCGCTAAATTG
ATGGGTTCGATTCGCCATACGAGATCAAATTAATCTCAGAGAAAAATTTATATTGCCCTTGATGAA
CATGTTGAGTTTTAGAGCTAGAAATAGCAAGTTAAAATAAGGCTAGTCCGTTATCAACTTGAAAAA
GTGGCACCGAGTCGGTGCTTTTTTTTTTTTTTGTTTTTTATGTCT**GGATCC**

SacI restriction site
BamHI restriction site

SNR52 RNA pol III promoter from *C. parapsilosis*
Guide B targeting *ADE2*
gRNA scaffold
tRNA terminator sequence from *S. cerevisiae SUP4*

Fig. S6 Sequence of GAPDH-HH-ADE2B-HDV

GAGCTCGTACTTTGGTGTAACTGGGGTATTTTGCCCGGTGATAAAATAGAAGGGACGTGGGAAT
AGTTGTGAGTGGAAATTGTTGATCCGATATGTTGAGGAAAAGTCCGATGTGAATCGAATGATAGA
GGTATTACTTTAAATAAGGAAAAGGGTGTCGGTTGGGTTGTGAGTTTGTGCGTCTAAGCATTGTC
AGTCGTCCGTCATCATTTGCAGACCGAACCCCATTGAGCTAGGGTCGTAGAACCACGTAGGTAG
GGTGAGATTAGCAGTGCTGTATATCCGATGGACGATTATGACCCGGCGGTATGACTTTGTTGCA
GCGCGAGTGTGCACTATTGTACAAAAAGCAAAATTAAAAATGAATATATGCTACCATATGATTTTT
AACTACATACGTTCTACTCTCTTCCGTATTACAAACAAACAACTGACACAAAAGAAGACAACAACT
AATGAGAAAAATGTCACGTTGACCCGAAATCTTTTTCTCCTTTCTTTGCCATTTTCTGATTATTTAA
CCAATTGAAATTTCTCAACAAATTTTTAAATACAATTCTTCTTCCTTCCTTCCTTCCTTCTTTCTTTC
TTTCTTTTCCAAATTCA**AAGGGCCTGATGAGTCCGTGAGGACGAAACGAGTAAGCTCGTC**GCCC
TTGATGAACATGTTGAGTTTTAGAGCTAGAAATAGCAAGTTAAAATAAGGCTAGTCCGTTATCAAC
TTGAAAAAGTGGCACCGAGTCGGTGCTTTT**GGCCGGCATGGTCCCAGCCTCCTCGCTGGCGCC**
GGCTGGGCAACATGCTTCGGCATGGCGAATGGGACCTATCCGTGAAGTTATAAGAAGGCAATT
AAGAGCTAGCTAGCTAGTCTTTGGTTTTCTTTTGGTTTTATATATTTTTCGTATATGAGTTTATAAAT
AAAG**GGATCC**

SacI restriction site
BamHI restriction site

CPAR2_808670 (*GAPDH*) RNA pol II promoter from *C. parapsilosis*
Hammerhead ribozyme
Hammerhead: 6 bp reverse complementary to the first 6 bp of guide B
Guide B targeting *ADE2*
gRNA scaffold
Hepatitis Delta Virus ribozyme
Terminator sequence from *C. parapsilosis CPAR2_808670*

Supplementary material for Figs 1-3

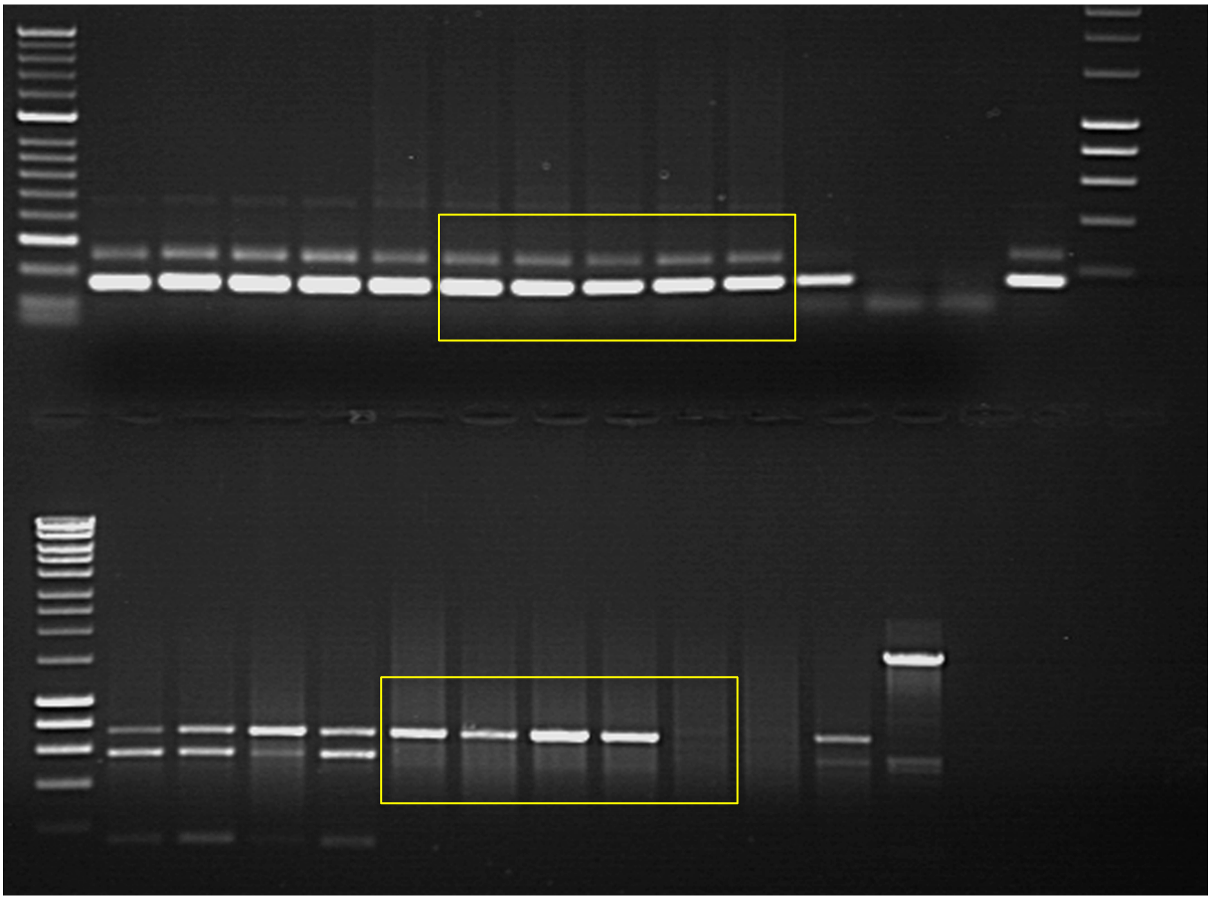

Fig. 1B. Entire gel for Fig. 1B. The box on the top shows the region used in the lower panel of Fig. 1B, and the box on the bottom shows the region used in the upper panel.

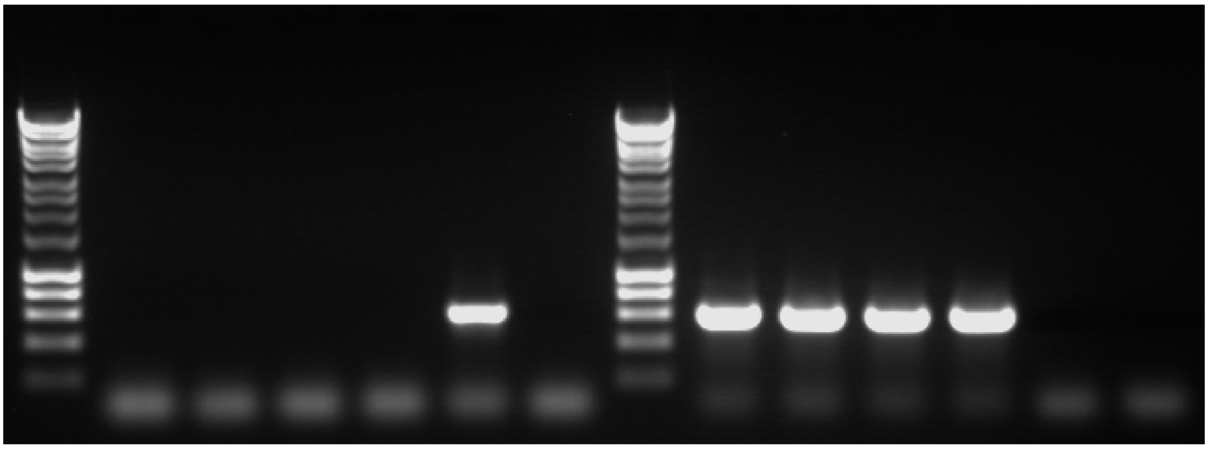

Fig 2C. Entire gel was used in Fig. 2C

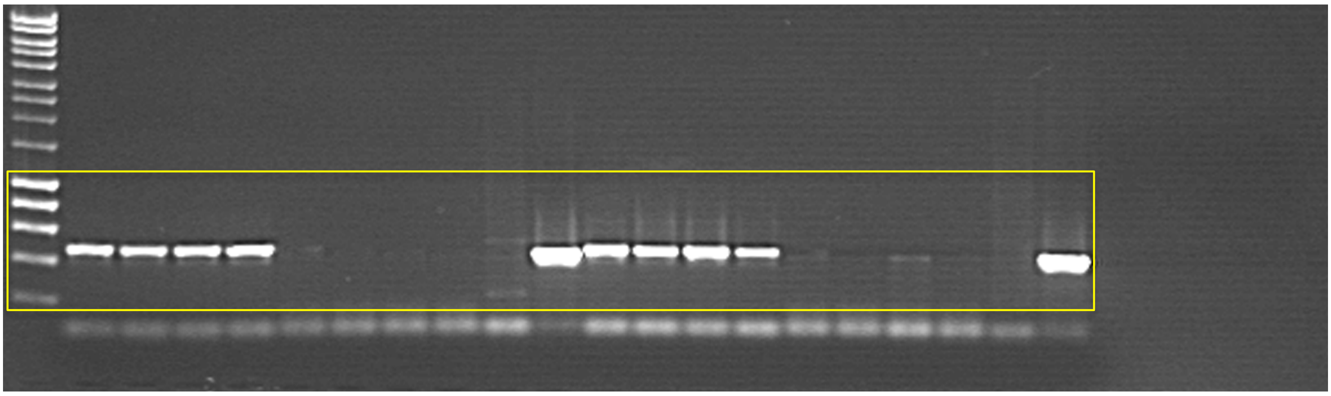

Fig 3E. Entire gel for Fig. 3E. The region used in the figure is highlighted with a box.

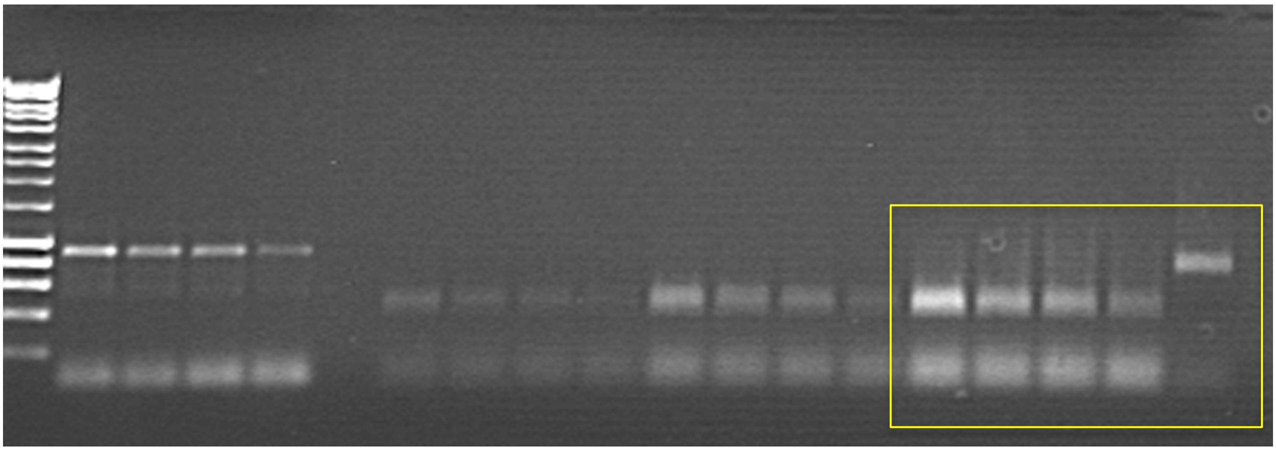

Fig 3F. Entire gel for Fig. 3F. The region used in the figure is highlighted with a box.

Table S1. Oligonucleotide sequences used in this study.

Name	Sequence 5'-3'	Application
**CAS9**		
A-CpCAS9 gBlock1-Fw	GAGATCGGTACTTCGCGAAT	Amplification of gBlock1
B-CpCAS9 gBlock1-Rv	AAACCGTTCTTCTTTTCACC	Amplification of gBlock1
C-CpCAS9 gBlock2-Fw	TCAATTGCCAGGTGAAAAGA	Amplification of gBlock2
D-CpCAS9 gBlock2-Rv	TTCCATGGAGTGATAGTTTC	Amplification of gBlock2
E-CpCAS9 gBlock3-Fw	AAAGTCTGAAGAAACTATCA	Amplification of gBlock3
F-CpCAS9 gBlock3-Rv	TCTGCACTCGTCGGTCCCGG	Amplification of gBlock3
F-CpCAS9 gBlock3-Rv	CAGTGAATTGGAGATCGGTA	Amplification of gBlock4
H-CpCAS9 gBlock4-Rv	CAACGTGCTTAGTGATTTGT	Amplification of gBlock4
I-CpCAS9 gBlock5-Fw	TTGAAACTAGACAAATCACT	Amplification of gBlock5
J-CpCAS9 gBlock5-Rv	CTTCAACTTCTTAGACTTAC	Amplification of gBlock5
K-CpCAS9 gBlock6-Fw	GTTGAAAAGGGTAAGTCTAA	Amplification of gBlock6
L-CpCAS9 gBlock6-Rv	CGCTTGCACGCCTCTGCACT	Amplification of gBlock6
**Generation of pSAT1**		
CpCAS9_GA_1	CAGTGAATTGGAGATCGGTACTTCGCGAATGCG TCGAGATACCGGTATGGACAAGAAGTA	Amplification of gBlocks1+2+3
CpCAS9_GA_2	TGTCTTCCTTGAAAGTCAAA	Amplification of gBlocks1+2+3

CpCAS9_GA_3	CAATTGATCCACGACGACTCTTTGACTTTCAAG GAAGACATCCAAAAGGCTCAAGTTTCT	Amplification of gBlocks4+5+6
CpCAS9_GA_4	CACGCCTCTGCACTCGTCGGTCCCGGCATCCG ATGGATCCTTAAACCTTTCTCTTCTTCT	Amplification of gBlocks4+5+6
TEF1p_AgeI_Fw	AGATCGGTACTTCGCGAATGCGTCGAGATAACC GGTACACCATCATTGATGATGAT	Amplification of CpTEF1 promoter
TEF1p_AgeI_Rv	AACCGATAGAGTACTTCTTGTCCATACCGGACC GGTCACTCTCTAAGTCTATGATG	Amplification of CpTEF1 promoter
TEF1t_BamHI_Fw	CTGACCCAAAGAAGAAGAGAAAGGTTTAAGGGA TCCATTATGTAGGTGAATGGTGA	Amplification of CpTEF1 terminator
TEF1t_BamHI_Rv	GCACTCGTCGGTCCCGGCATCCGATGGATCGG ATCCTATAACCGATGGAAGCTTGA	Amplification of CpTEF1 terminator
GA_cpARS_Fw	GGGTTCCGCGCACATTTCCCCGAAAAGTGCCAC CTGACGTATAGGCGTATCACGAGGCCC	Amplification of ARS7
GA_cpARS_Rv	ATTTTTATAGGTTAATGTCATGATAATAATGGTTT CTTAGTTACAGACAAGCTGTGACCG	Amplification of ARS7
SapI_CaSAT1-Fw	GCGCTACGCTACCGCTCTTCGGATTAGTAGCCT AGACATCAGCAGCTGCTTGGA	Amplification of CaACT1-SAT1
SapI_CaSAT1-Rv	TACTAGCGATGAGCCGAAGAGCACTGACTAGAG ATCCGGTTTCATGTACCAACTCAC	Amplification of CaACT1-SAT1
**RT-PCR**		
CpACT1_F	GAAGCTTTGTTCCGTCCAGC	Amplification of 150 bp in *ACT1* open reading frame
CpACT1_R	TGATGGAGCCAAAGCAGTGA	Amplification of 150 bp in *ACT1* open reading frame
**Generation of pUC57 based plasmids.** GA = Gibson Assembly		
GA7_pUC57F	TTTAGAGCTAGAAATAGCAAGTTAAAATAAGGCT AGTCC	Linearization of pUC57_RIBO_sgRNA for GA with HH-sgRNA insert
GA7_pUC57R	GAATTTGGAAAAGAAAGAAAGAAAGAAG GAAGGAAGG	Linearization of pUC57_RIBO_sgRNA for GA with HH-sgRNA insert
New_HHsgRNAD_TOP	TTCTTTCTTTTCCAAATTCACAATGTCTGATGAG TCCGTGAGGACGAAACGAGTAAGCTCGTC	Generation of the insert HH-sgADE2-D by primer extension
New_HHsgRNAD_B	GCTATTTCTAGCTCTAAAACCTTCCGTCATATGC	Generation of the insert

OT	CAATGTGACGAGCTTACTCGTTTCGT	HH-sgADE2-D by primer extension
GA2_URA3_sgRNA358_FWD	TTCTTTCTTTTCCAAATTCAATTTGTCTGATGAGTCCGTGAGGACGAAACGAGTAAGC	Generation of the insert HH-sgURA3-358 by primer extension
GA2_URA3_sgRNA358_RV	GCTATTTCTAGCTCTAAAACGTGACGCCATGTGCATTTGTGACGAGCTTACTCGTTTCGTCCTCA	Generation of the insert HH-sgURA3-358 by primer extension
GA2_URA3_sgRNA195_FWD	TTCTTTTCCAAATTCATCGATGCTGATGAGTCCGTGAGGACGAAACGAGTAAGCTCGTCCATCG	Generation of the insert HH-sgURA3-195 by primer extension
GA2_URA3_sgRNA195_RV	GCTATTTCTAGCTCTAAAACCGTACGAGAAGTCATCGATGGACGAGCTTACTCGTTTCG	Generation of the insert HH-sgURA3-195 by primer extension
CP101060_HHsgRNAbTOP	TTCTTTCTTTTCCAAATTCACCACTCCTGATGAGTCCGTGAGGACGAAACGAGTAAGCTCGTC	Generation of the insert HH-sgCP101060-B by primer extension
CP101060_HHsgRNAbBOT	GCTATTTCTAGCTCTAAAACCCTTCCTAAACTTGCCACTCGACGAGCTTACTCGTTTCGT	Generation of the insert HH-sgCP101060-B by primer extension
**Generation of pSNR and pRIBO plasmids.** GA = Gibson Assembly		
GA2_pSAT1_F	CAGTGAATTGGAGATCGGTACTTCGTTAAGATATAGTTATCGGTTTTAATTC	Amplification of SNR-ADE2 insert for GA into NruI-digested pSAT1
GA2_pSAT1_R	GTGTACCGGTATCTCGACGCATTCGAGACATAAAAAACAAAAAAAAAAAAAAG	Amplification of SNR-ADE2 insert for GA into NruI-digested pSAT1
GA_pSAT1_F	CAGTGAATTGGAGATCGGTACTTCGGTACTTTGGTGTAACTGG	Amplification of GAPDH-HH-ADE2-HDV-t insert for GA into NruI-digested pSAT1
GA_pSAT1_R	GTGTACCGGTATCTCGACGCATTCGCTTTATTTATAAACTCATATACGAAAAATATATAAAAC	Amplification of GAPDH-HH-ADE2-HDV-t insert for GA into NruI-digested pSAT1
pRIBO_URA3_FWD	AATTGGAGATCGGTACTTCGGTACTTTGGTGTAACTGG	Amplification of GAPDH-HH-URA3-HDV-t insert for GA into NruI-digested pSAT1
pRIBO_URA3_REV	CCGGTATCTCGACGCATTCGCTTTATTTATAAAC	Amplification of GAPDH-

	TCATATACGAAAAATATATAAAAC	HH-URA3-HDV-t insert for GA into NruI-digested pSAT1
**Repair templates.** The mutated PAM sequence is shown in bold, and the designed stop codons are colored in red. The guide sequence is highlighted in grey. The barcode sequences inserted at the *CPAR2_101060* and *ADE2* loci are highlighted in cyan and the KpnI recognition site in yellow. RT = Repair Template		
RepTempB_Top	GATGCTCCGCAATCACCAGCCAAGCAAATCAATGCCCTTGATGAACATGTTGA**TTA**ATAG	Generation of RTB by primer extension (sgADE2-B)
RepTempB_Bot	GGCAATAGATTCATAATCAGTGAACTATT**TAA**TCAACATGTTCAT	Generation of RTB by primer extension (sgADE2-B)
RepTempD_Top	GCGTATGGATATCCATACATGTTAAAATCGAGAACATTGGCATATGACGGAAG**ATA**ATAG	Generation of RTD by primer extension (sgADE2-D)
RepTempD_Bot	CATATGATTCATCTTTGACAACGAACTAT**TAT**CTTCCGTCATATG	Generation of RTD by primer extension (sgADE2-D)
RepTemp_sgRNA358_short_FWD	GTCTATAAGATTGCCAAATGGGCAGATTTGACAAATGCACATGGCGTCAC**TTA**ATAG	Generation of RT358 by primer extension (sgURA-358)
RepTemp_sgRNA358_short_REV	GAGCTCCTTCTTTCAATCCTTTAACCACCCCCTAT**TAA**GTGACGCCATGTGCATTTG	Generation of RT358 by primer extension (sgURA-358
RepTemp_sgRNA195_short_FWD	CATATGCTTGGTCAAAACACATATCGACATCATCGATGACTTCTCGTACG**TAG**TAAG	Generation of RT195 by primer extension (sgURA-195)
RepTemp_sgRNA195_short_RV	CTTCGATAGCTCGAGTAGGGGAAGAATAGTTA**CTA**CGTACGAGAAGTCATCGATG	Generation of RT195 by primer extension (sgURA-195)
CP101060_RTb_Bar_T	TTTGGGAATTCAAACATCTGAGTGGCAAGTTTAGGAAG**TAA**TAGTGGCCGCATTTCGCAGATGTGGTACC	Generation of RTB by primer extension (sgCPAR2_101060)
CP101060_RTb_Bar_B	GAGCTCGATCTCCGTTTGATATAAACTAATGAACTTTCGGTACCACATCTGCGAAATGCGGCCACTA**TTA**	Generation of RTB by primer extension (sgCPAR2_101060)
RT_DEL_TOP	CCCTGAAACAGTCTCTCAGTTCCATTTACTTCGCATCACCCCCGTTCGTTAATACGTGAT	Generation of RT_DEL by primer extension (sgADE2-B/D)

RT_DEL_BOT	ATAAATTTAATGTATAATAACTATAACTTTAGC ACAATTTATCACGTATTAACGAACGGG	Generation of RT_DEL by primer extension (sgADE2-B/D)
**Colony PCR**		
wtADE2B_F	GCCCTTGATGAACATGTTGATGGATCC	Detection of native sequence in *ADE2* locus
mutADE2B_F	GCCCTTGATGAACATGTTGATTAATAG	Detection of mutated sequence in *ADE2* locus (due to RTB)
wtADE2D_F	GGCATATGACGGAAGAGGTAAT	Detection of native sequence in *ADE2* locus
mutADE2D_F	GGCATATGACGGAAGATAATAG	Detection of mutated sequence in *ADE2* locus (due to RTD)
ADE2_FWD	CTTCGCATCACCATGGACAG	Sequencing of the *ADE2* locus
ADE2_REV	CAAATATACCACAACCAGGG	Sequencing of the *ADE2* locus/common primer
URA3_mutant_FWD	GGTTGACAAGCTTGGACCTTAC	Sequencing of the *URA3* locus
URA3_mutant_REV	AACCCCAGGAGTCATGATTACC	Sequencing of the *URA3* locus
CP101060_WT_F	GTTTAG GAAGGGGGATGAAA	Detection of wildtype sequence at CP101060
CP101060-F	ATGTCGAAAAAGAGTCAGGG	Sequencing CP101060
CP101060_WT_R	GTTGCGGCTGTAGTTGAG	Common primer at CP101060
CP101060_MUT_F	GTTTAGGAAGTAATAGTGGC	Detection of mutated sequence at CP101060
CpCAS9A_FWD	TCAATTGCCAGGTGAAAAGA	Evaluation of random *CAS9* genomic integration
CpCAS9A_REV	TTCCATGGAGTGATAGTTTC	Evaluation of random *CAS9* genomic integration
DEL_FWD	ATTCTTAATTCATTCAAAGC	Verification of *ADE2* deletion mutants.
DEL_REV	AGAGAGGGTGATCACGTACA	Verification of *ADE2*

		deletion mutants.

Sequencing of pSAT1			
Seq_1	GCCTCTTCGCTATTACGCCA	Seq_6.1	TTCGACTCTCCAACTGTTGC
SeqFw1	GAAATCTCTGGTGTTGAAGAC	Seq_6.2	TCAATTCGTTGTAAACAGTG
SeqRv1	CAACTGGGTGTTCCTTCAAGA	Seq_7	ATTGTTGGTTAAGTTGAACA
Seq_1.1	GGAAACGGGATTGACTGCAA	Seq_7.1	TGTTTAAAATCCTGTTCAAC
SeqFw2	TGGGTATCACTATCATGGA	Seq_7.2	ACTGCCGTTGGAATATCTTC
Seq_2	TGATAGAGAGAAGAGCCCCT	Seq_8	TTGAAAGTCAAAGAGTCGTC
SeqRv2	GATTGGTGGATCAAAGTAGCGT	Seq_9	ATCTGGTAAGACTATCTTGG
Seq_3	ACTACACTTGCAAGACGATG	Seq_9.1	GCAAGTAGTACAAGTACAAC
Seq_3.1	TGACTTGTTCTTGGCTGCTA	Seq_10	CGTTCAAGTAAGCGTCGTGA
SeqFw3	CTTGGGTGCTCCAGCTGCTT	Seq_10.1	CTAACAGTAGCGAAGTCTCT
Seq_4	TCACCTTCGATCAAGAAGTG	Seq_11	AGTTGATCAGAGAAGTTAAG
Seq_4.1	CAGCCAAGTCGAAGTTAGAC	Seq_12	TGTTCAACGAACAATTGCTT
Seq_5	ACGAAGTTGCTTACCACGAA	Seq_12.1	TGAACTTAATCAGAATATAC
Seq_5.1	TGTACTTGTACTACTTGCAA	Seq_12.2	GCGTCAAGGCTAATCCTATAC
Seq_6	CAAGAATGGGTAGAAGTCTT	Seq_13	ATTGGCTTTGCCATCTAAGT
		Seq_14	CAATACGCAAACCGCCTCTC

Table S2. Strains used in this study.

**a. Clinical isolates of *C. parapsilosis***			
**Strain**	**Source**	**Location**	**Reference**
CLIB214	Feces	Puerto Rico	Type strain
CDC165	Blood	Mississippi, USA	[1]
CDC167	Blood	Mississippi, USA	[1]
CDC173	Blood	Mississippi, USA	[1]
CDC179	Blood	Mississippi, USA	[1]
CDC317	Hands	Mississippi, USA	[1]
73/037	Vagina	Leeds, United Kingdom	[2]
73/107	Mouth	London, United Kingdom	[2]
74/046	Aortic valve	Leeds, United Kingdom	[2]
90-137	Orbital tissue	San Jose, California, USA	[2]
81/040	Toe space	London, United Kingdom	[2]
81/042	Ear	Leeds, United Kingdom	[2]
81/253	Nail	London, United Kingdom	[2]
02-203	Blood	Bergamo, Italy	[2]
103	Anus	London, United Kingdom	[2]
J931845	Unknown	Japan	[2]
J931058	Nail	Belgium	[2]
J950218	Unknown	USA	[2]

J951066	Nail	Korea	[2]
J961250	Nail	Lisbon, Portugal	[2]

**b. Mutant strains generated**				
**Strain Name**	**Parent Strain**	**Genotype**	**gRNA**	**Repair template (RT)**
CRISPR_LL1	CLIB214	$ade2^{-}/ade2^{-}$	sgADE2-B	RTB
CRISPR_LL2	CLIB214	$ade2^{-}/ade2^{-}$	sgADE2-B	RTB
CRISPR_LL3	CLIB214	$ade2^{-}/ade2^{-}$	sgADE2-D	RTD
CRISPR_LL4	CLIB214	$ade2^{-}/ade2^{-}$	sgADE2-D	RTD
CRISPR_LL5	CLIB214	$ura3^{-}/ura3^{-}$	sgURA3-358	RT358
CRISPR_LL6	CLIB214	$ura3^{-}/ura3^{-}$	sgURA3-358	RT358
CRISPR_FZ1	90-137	$ura3^{-}/ura3^{-}$	sgURA3-358	RT358
CRISPR_FZ2	90-137	$ura3^{-}/ura3^{-}$	sgURA3-358	RT358
CRISPR_LL7	CLIB214	$ade2^{-}/ade2^{-}$, $ura3^{-}/ura3^{-}$	sgADE2-D + sgURA3_358	RTD + RT358
CRISPR_LL8	CLIB214	$ade2^{-}/ade2^{-}$, $ura3^{-}/ura3^{-}$	sgADE2-D + sgURA3_358	RTD + RT358
CRISPR_ST1	CLIB214	$cp101060^{-}/cp101060^{-}$	sgCP101060-B	CP101060_RTb_Bar
CRISPR_ST2	CLIB214	$cp101060^{-}/cp101060^{-}$	sgCP101060-B	CP101060_RTb_Bar

CRISPR_ST3	90-137	*cp101060*$^-$/*cp101060*$^-$	sgCP101060-B	CP101060_RTb_Bar
CRISPR_ST4	90-137	*cp101060*$^-$/*cp101060*$^-$	sgCP101060-B	CP101060_RTb_Bar
CRISPR_ST5	90-137	*ade2Δ::ade2Δ*	sgADE2-B	RT_DEL
CRISPR_ST6	90-137	*ade2Δ::ade2Δ*	sgADE2-D	RT_DEL

References

1 Kuhn, D. M. *et al. Candida parapsilosis* characterization in an outbreak setting. *Emerg. Infect. Dis.* **10**, 1074-1081 (2004).

2 Tavanti, A., Davidson, A. D., Gow, N. A., Maiden, M. C. & Odds, F. C. *Candida orthopsilosis* and *Candida metapsilos*is spp. nov. to replace *Candida parapsilosis* Groups II and III. *J. Clin. Microbiol.* **43**, 284-292 (2005).

Table S3. Plasmids generated in this study.

Name	Description
**pUC57 plasmids used for the generation of pSAT1**	
pUC57_CAS9	Codon optimized CAS9 in pUC57
pUC57_pCAS9t	Codon optimized CAS9 flanked by CpTEF1 regulatory regions in pUC57
pUC57_ARS_pCAS9t	ARS7 autosomal replicating sequence in pUC57-pCAS9-t
**pUC57 plasmids used for the replacement of the HH-guide RNA insert**	
pUC57_CpSNR52p_ADE2_sgRNAB	SNR-ADE2B in pUC57
pUC57_HH_HDV_ADE2_sgRNAB	GAPDH-HH-ADE2B-HDV in pUC57
pUC57_HH_HDV_ADE2_sgRNAD	GAPDH-HH-ADE2D-HDV in pUC57
pUC57_HH_HDV_URA3_sgRNA195	GAPDH-HH-URA3-195-HDV in pUC57
pUC57_HH_HDV_URA3_sgRNA358	GAPDH-HH-URA3-358-HDV in pUC57
pUC57_HH_HDV_CP101060_sgRNA_B	GAPDH-HH-CP101060-B-HDV in pUC57
**pSAT1-based plasmids**	
pSAT1	Contains *CAS9* under the control of CpTEF1p, Nat^R, and ARS7 for propagation in *C. parapsilosis*.

pSNR-ADE2-B	pSAT1 plasmid including SNR52 -sgRNA B directed against *C. parapsilosis ADE2.*
pRIBO-ADE2-B	pSAT1 plasmid containing *GAPDH*-HH-sgRNA B-HDV directed against *C. parapsilosis ADE2*. The gRNA is released by ribozyme cleavage.
pRIBO-ADE2-D	pSAT1 plasmid containing *GAPDH*-HH-sgRNA D-HDV directed against *C. parapsilosis ADE2*. The gRNA is released by ribozyme cleavage.
pRIBO-URA3-195	pSAT1 plasmid containing *GAPDH*-HH-sgRNA195 -HDV directed against *C. parapsilosis URA3*. The gRNA is released by ribozyme cleavage.
pRIBO-URA3-358	pSAT1 plasmid containing *GAPDH*-HH-sgRNA358 -HDV directed against *C. parapsilosis URA3*. The gRNA is released by ribozyme cleavage.
pRIBO-CP101060-B	pSAT1 plasmid containing *GAPDH*-HH-sgRNA B-HDV directed against *C. parapsilosis CPAR2_101060*. The gRNA is released by ribozyme cleavage.

Supplementary methods: Instructions for using CRISPR-Cas9 in *C. parapsilosis*

Design a new sgRNA construct:

The starting point is any pUC57_RIBO_sgRNA construct. The sgRNA is introduced into this plasmid first and then moved to pSAT1 because of the lack of suitable restriction sites in the latter plasmid.

The pUC57_RIBO_sgADE2-B construct is used below as an example (Table S3).

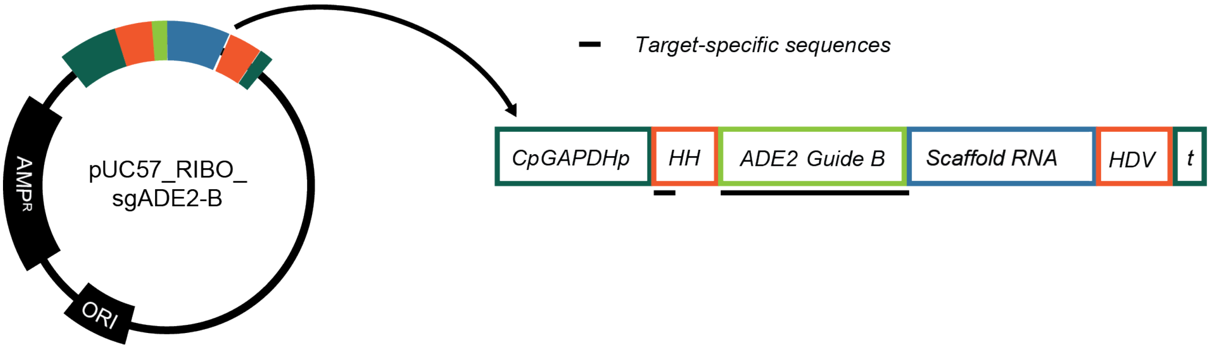

In order to change the target gene, two sequences must be changed:

A. The 6 bp at the 5' end of the HH ribozyme must be complementary to the first 6 bp from the new target sequence, because it forms part of the first stem of the ribozyme.
B. The existing sgRNA target sequence must be replaced with a new sgRNA (20 bp)

This is accomplished by replacing a 103 bp fragment, encompassing the HH region (orange) and the sgRNA (green).

1. First design a new 20 base short guide RNA, immediately upstream of a PAM site.

2. Next design two overlapping oligonucleotides, TOP and BOTTOM as shown below. The TOP oligo contains 6 bases (N') that are complementary to the first 6 bases (N) of the sgRNA in green. The BOTTOM oligo contains the new sgRNA including 6 bases that are complementary to the N' bases (N) and the remaining 14 bases of the guide RNA (n). The sequences that change in each construct are underlined below.

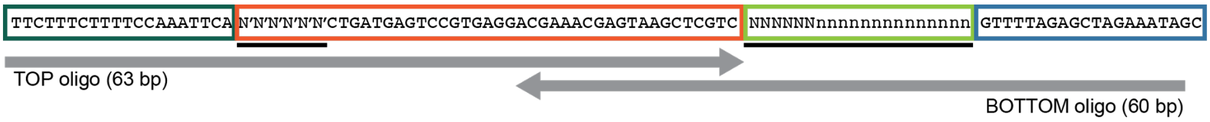

3. Use primer extension to produce the 103 bp fragment.

4. Linearize the plasmid by PCR using primers GA7_pUC57F and GA7_pUC57R. These primers overlap with the 5' and 3' ends of the 103 bp fragment by 18 bp (next page, left). Insert the 103 bp fragment into pUC57_RIBO_sgRNA by Gibson Assembly.

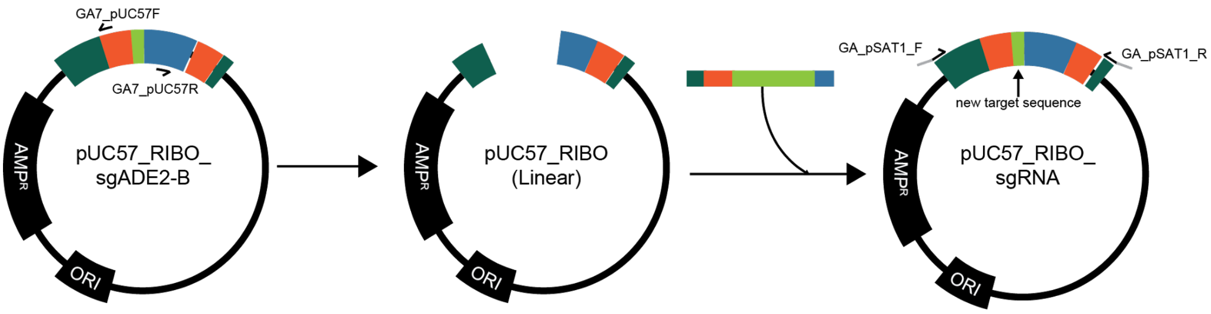

5. Next move the entire 911 bp GAPDH-HH-sgRNA-HDV-t cassette into pSAT1. First amplify the cassette by PCR using primers GA_pSAT1_F and GA_pSAT1_R. These include 23 bp (indicated in grey) that overlap with the region around the NruI site in pSAT1.

6. Digest pSAT1 with NruI, and introduce the GAPDH-HH-sgRNA-HDV-t by Gibson assembly, generating the final construct, pRIBO-sgRNA.

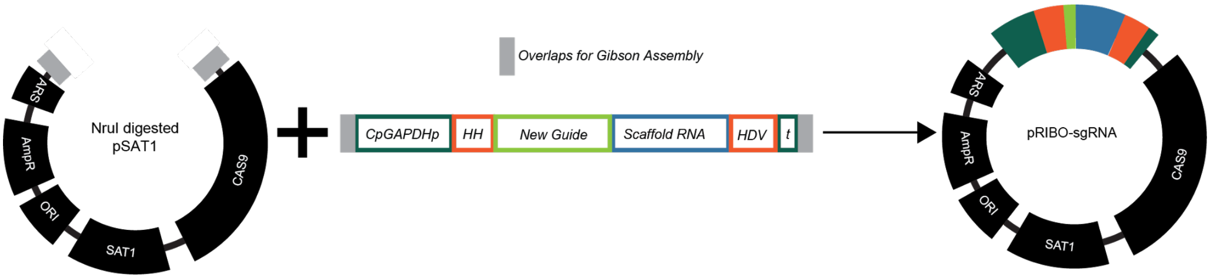

Generation of a repair template:

The 84 bp repair template is generated by primer extension of two oligonucleotides, TOP and BOTTOM. ADE2-B is shown here as an example. The oligonucleotides overlap at their 3' ends and both encoding the desired mutations. In the figure below, the target sequence is highlighted in grey, the PAM site is in bold and the mutated nucleotides are in red.

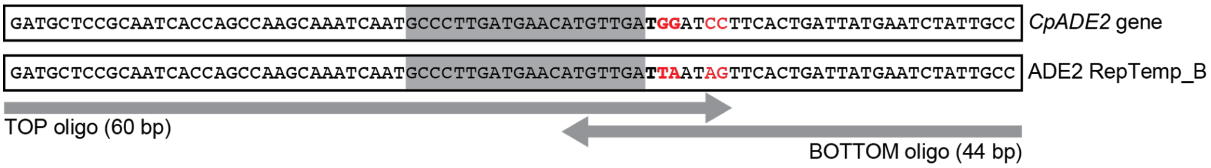

To insert additional sequences at the CPAR2_101060 locus, the same principle was applied. The repair template is larger (108 bp) due to the inclusion of a 20 bp tag and a KpnI site. Below, the tag is highlighted in blue and the KpnI site in green.

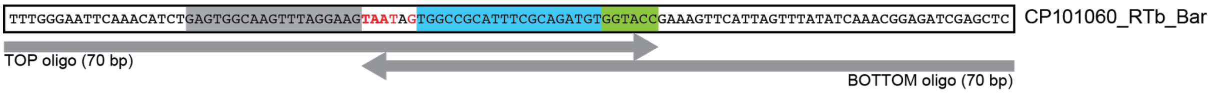

Transformation

Transform *C. parapsilosis* strains using the lithium acetate method with 5 µg pRIBO-sgRNA alone or together with 5 µg of the appropriate repair template. Select transformants on YPD plates containing 200 µg/mL nourseothricin at 30°C.

Include pSAT1 as a control. This usually yields >10 times more nourseothricin resistant transformants than the pRIBO constructs. The transformation efficiency varies from less than 10 colonies to more than 1000.
